# Light-driven radical catch-and-release with BODIPY photocages

**DOI:** 10.1039/d6sc01848c

**Published:** 2026-04-27

**Authors:** Anna Poryvai, Anna Vasiļevska, Karolína Bangievská, Ján Tarábek, Sandrine Gerber-Lemaire, Tomáš Slanina

**Affiliations:** a École Polytechnique Fédérale de Lausanne, SB ISIC SCI-SB-SG Station 6 CH-1015 Lausanne Switzerland anna.poryvai@epfl.ch; b Institute of Organic Chemistry and Biochemistry of the Czech Academy of Sciences Flemingovo Náměstí 542/2 Prague 6 160 00 Czech Republic; c Department of Organic Chemistry, Faculty of Science, Charles University Albertov 6 128 00 Prague 2 Czech Republic

## Abstract

Photocages release payloads upon light irradiation and are widely used for spatiotemporal control in chemical biology and materials science. Although payload release is almost universally described as a heterolytic process, homolytic pathways that generate radicals can interfere and produce unintended off-target effects. If controlled, radical photorelease would open new avenues for applications such as polymerization, however the molecular factors that govern this process in photocages remain unknown. Here, we investigate how photophysics and payload identity influence heterolytic *vs.* homolytic reactivity in BODIPY photocages. We find that high fluorescence quantum yields correlate with efficient homolytic cleavage, enabling reversible radical *catch-and-release*: light-assisted capture of radical payloads followed by clean photorelease under green light illumination. This radical release can be suppressed by the introduction of iodide or boron-methyl substituents which promote intersystem crossing. We achieve the highest photorelease quantum yield reported to date for green-light-driven radical generation (*Φ*_r_ = 0.5%), surpassing heterolytic carboxylate uncaging. We further exploit this reactivity in Type I photoinitiation of RAFT polymerization, yielding fluorescently labelled polymers with defined dispersity. This work establishes a structure–reactivity framework for predictable light-controlled radical generation, enabling mitigation of off-target radical effects and opening new avenues for late-stage photochemical payload installation.

## Abstract 2

BODIPY photocages, long considered heterolytic, undergo efficient homolytic cleavage—enabling predictable, green-light-driven radical payloads installation and photorelease.

## Graphical abstract

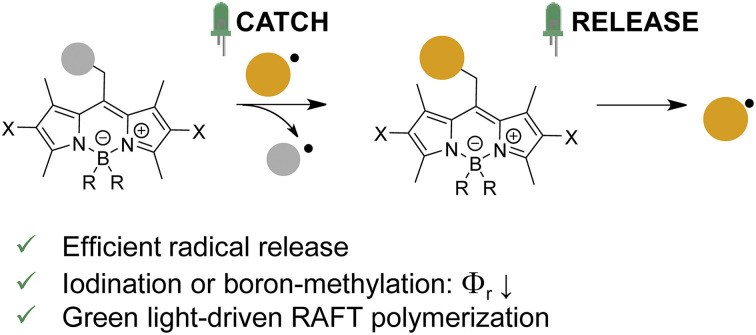

## Introduction

**Scheme 1:**
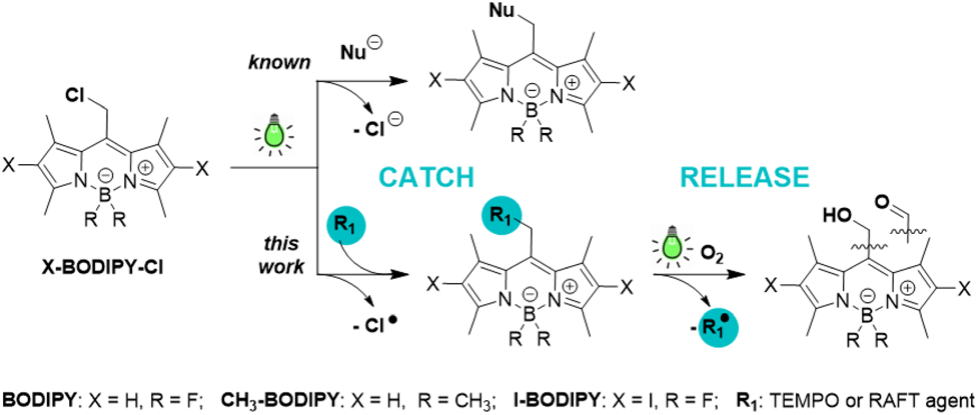
*Catch-and-release* photoreactivity of BODIPY–Cl.

Photocages are photoremovable protecting groups that temporarily mask active substrates through covalent bonds and release them upon irradiation.^1,2^ This capability enables precise spatial and temporal control over chemical and biological processes. In the majority of organic photocages, payload release is thought to proceed *via* heterolytic bond cleavage, yielding an anionic payload and a cationic cage (Scheme 1).^1,2^ In contrast, homolytic bond cleavage leading to radical payloads remains rare. Critically, the parameters that govern pathway selection—whether a given photocage produces ions or radicals upon irradiation—remain unknown. The absence of photocage design principles restricts predictive control over photocage reactivity, hinders rational design for radical-sensitive chemical and biological environments, and hinders the mitigation of undesired radical side effects.

Only a limited number of photocages have been shown to release radical payloads, namely nitric oxide,^3–7^ nitroxyl radicals,^8–19^ bromine- and sulfur-based groups.^20–23^ Homolytic release, however, interferes with heterolytic pathways, preventing clean and predictable radical generation.^8,15,20–23^ Recently, coumarin-based photocages were shown to favor homolytic release of bromine- and sulfur-centered payloads,^20,21^ while our prior work on BODIPY photocages revealed partial radical release of acetate and thiodansyl groups.^22,23^ Collectively, these studies underscore the need for rational photocage design principles that enable predictable pathway selection. There is also a need for strategies that enable easy, efficient late-stage payload installation on the photocage. Current approaches often rely on multi-step organic synthesis to install payloads, resulting in low yields and restricting access to sensitive compounds. A versatile *catch-and-release* approach, where a payload can be photochemically installed at a late stage in the synthesis (“*catch*”) and selectively photocleaved (“*release*”) would address this gap directly.

Here, we report that green-light-activatable BODIPY photocages can be directed to highly controllable reversible homolytic bond cleavage, enabling a radical *catch-and-release* process (Scheme 1). Using Cl and a persistent radical TEMPO payloads, we demonstrate (i) homolytic BODIPY–Cl bond cleavage to generate a BODIPY radical, (ii) *catch*: capture of this radical by TEMPO or RAFT agent, and (iii) *release*: efficient photorelease of the captured radical. Furthermore, we exploit this reactivity for living reversible addition–fragmentation chain transfer (RAFT) and free radical (FR) chain polymerizations of isobornylacrylate (IBOA). Together, these results establish that BODIPY photocage reactivity can be directed toward efficient homolytic pathways and provide design principles for predictable light-controlled radical *catch-and-release*.

### Heterolytic *vs.* homolytic reactivity

**Chart 1:**
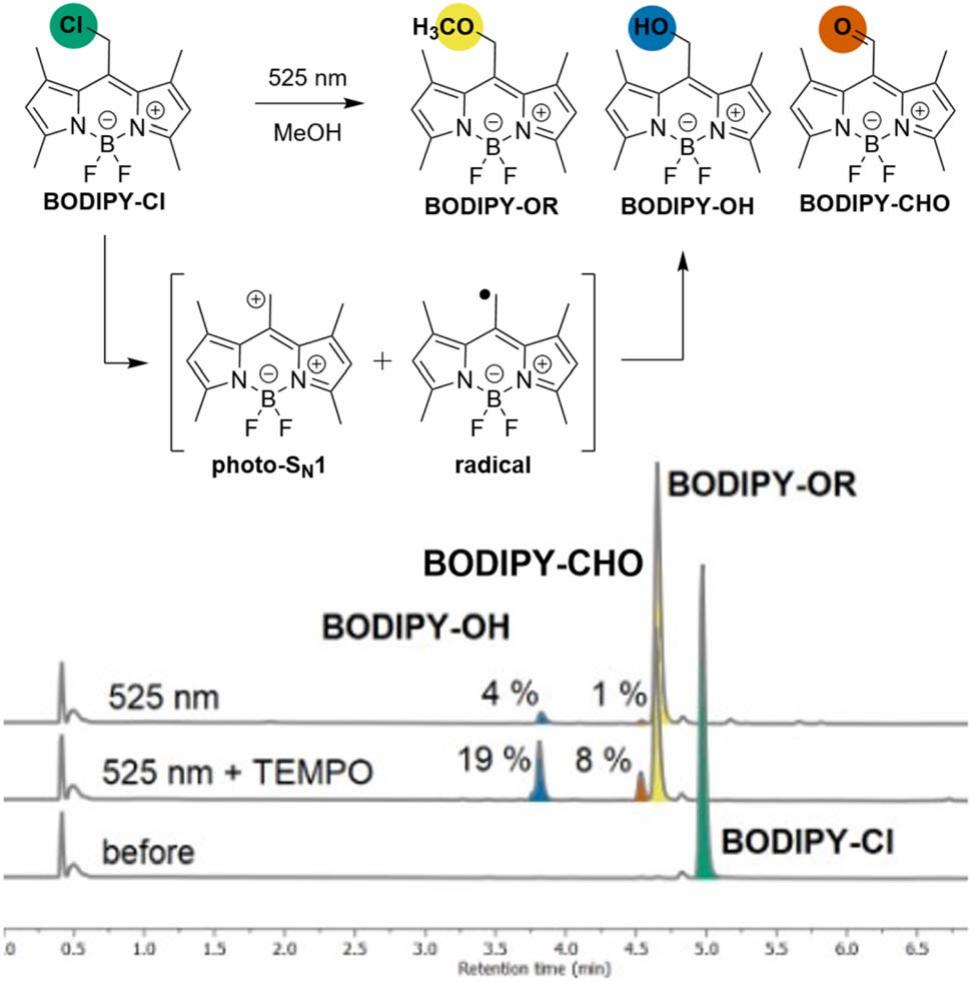
Reactivity of BODIPY–Cl in MeOH and corresponding traces from LC-MS analysis of solutions of BODIPY–Cl in MeOH with or without TEMPO (8 equiv.), before and after illumination with 525 nm light (8 min).

To investigate the interplay between ionic and radical photoreactivity, we examined BODIPY–Cl in the presence or absence of nucleophiles (methanol or *t*-butanol), which trap the reactive species formed through the heterolytic (photo-S_N_1) pathway, or TEMPO, which traps the intermediates of the homolytic (radical) pathway (Chart 1, Chapter S2-2). UV-Vis and LC-MS analysis of the reaction mixtures revealed that the irradiation of BODIPY–Cl in aerated anhydrous MeOH leads predominantly to the photo-S_N_1 product, BODIPY–OMe (Table S2-3: A), accompanied by traces of BODIPY–OH and an oxidation product, BODIPY–CHO^22^ (∼4% and 1%, respectively, Chart 1). Reaction in *t*-BuOH (as a bulky nucleophile) resulted in multiple products (Table S2-3: C, D, Fig. S2-9: C, D), while irradiation in PhH (a non-nucleophilic solvent) led solely to BODIPY–OH and BODIPY–CHO (Table S2-3: E, Fig. S2-10). Notably, the addition of 8 equiv. of TEMPO shifted the product distribution markedly, increasing the ratios of BODIPY–OH and BODIPY–CHO, to 19 and 8% in MeOH, respectively (Chart 1, Table S2-3: B, H). By contrast, a large excess of MeOH (180 equiv.) in PhH did not produce BODIPY–OMe; the photo-S_N_1 product was only observed when MeOH was present in large excess as co-solvent with PhH (280 000 equiv., 1 : 1 v/v, Table S2-3: G, H, I). Overall, these results demonstrate that the presence TEMPO efficiently diverts the photoreaction course from the heterolytic photo-S_N_1 pathway toward the radical pathway. We therefore hypothesised that irradiation of BODIPY–Cl generates a BODIPY-centred radical intermediate that is subsequently intercepted by TEMPO in a *catch-and-release* mechanism, with BODIPY–OH and BODIPY–CHO arising as by-products of the radical pathway. To probe this hypothesis, we next investigated the catch process directly.

### Catch

To confirm this hypothesis, we attempted to *catch* a TEMPO payload onto the BODIPY-methyl scaffold (Table 1). A concentrated solution of BODIPY–Cl with TEMPO was initially irradiated in a flask, providing BODIPY–TEMPO in a low yield, which was improved by performing the photoreaction in a flow reactor^24^ with the more uniform illumination of the reaction mixture (Table 1, entries 1, 2). Moving from benzene to toluene (PhCH_3_, Table 1, entry 3) and optimizing the reagents ratio and flow rate led to a significant yield increase (up to 87%, Table 1, entry 4).

**Table 1:** Optimization of the *catch* process of TEMPO by BODIPY–Cl

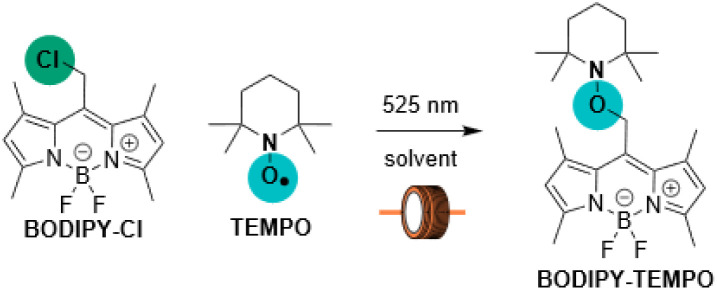					
Entry	*c* (BODIPY) [mM]	TEMPO [equiv.]	Solv.	Isol. yield [%]	Setup, flowrate [mL min^−1^]
1	20	2	PhH	9	Batch
2	4	9	PhH	52	Flow, 0.15
3	4	9	PhCH_3_	43	Flow, 0.05
4	4	11	PhCH_3_	87	Flow, 0.5

Boron-methylation and iodination of the BODIPY scaffold have previously been shown to significantly increase the efficiency of heterolytic photo-S_N_1 release in MeOH (*Φ*_r_ ∼ 2% for BODIPY–Cl *vs. Φ*_r_ ∼ 35% and 45% for CH_3_–BODIPY–Cl and I–BODIPY–Cl, respectively).^25^ We therefore investigated whether these structural modifications also enhance radical reactivity. Therefore, we studied the abovementioned chlorides as substrates for TEMPO *catch*. Addition of TEMPO to a methanolic solution of CH_3_–BODIPY–Cl resulted in higher conversion to the homolytic bond cleavage products (Fig. S2-14). Irradiation in PhH, however, led to a rapid decomposition of CH_3_–BODIPY–Cl with no chromophoric products (Fig. S2-14). I–BODIPY–Cl remained unreactive in PhH. High photo-S_N_1 reactivity was observed in MeOH with and without TEMPO additive (Fig. S2-16). Addition of a larger excess of TEMPO (34 equiv.) resulted in the negligible formation of I–BODIPY–OH alongside very efficient conversion to the photo-S_N_1 product I–BODIPY–OMe (Fig. S2-16).

**Chart 2:**
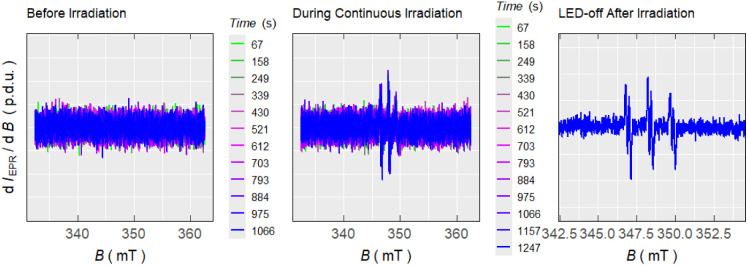
Evolution of EPR spectra recorded in time of solutions of BODIPY–Cl in PhH in the presence of *N-tert*-butyl-α-phenylnitrone spin trap, measured in dark (left), upon illumination with 525 nm light (middle), and after irradiation (right).

The intermediacy of the BODIPY-methyl radical was probed by electron paramagnetic resonance (EPR) spectroscopy, monitoring the appearance of the aminoxyl radical product of its addition to a *N-tert*-butyl-α-phenylnitrone spin-trap (Chart 2, Fig. S2-11). As a complementary experiment we studied the *catch* process upon irradiation of excess BODIPY–Cl and TEMPO in PhH, leading to the BODIPY–TEMPO conjugate, by monitoring the decrease of TEMPO concentration (Fig. S2-12). The EPR measurements of I–BODIPY–Cl with TEMPO showed no decrease in TEMPO concentration, further supporting the lack of photoreactivity observed by UV-Vis and LC-MS techniques (Fig. S2-17). These results corroborate our hypothesis of BODIPY–Cl undergoing homolytic bond cleavage upon irradiation, generating a BODIPY-methyl radical that is efficiently intercepted by TEMPO, thereby diverting the reaction pathway from photo-S_N_1 reactivity to productive radical trapping (Scheme 1).

### Release

**Chart 3:**
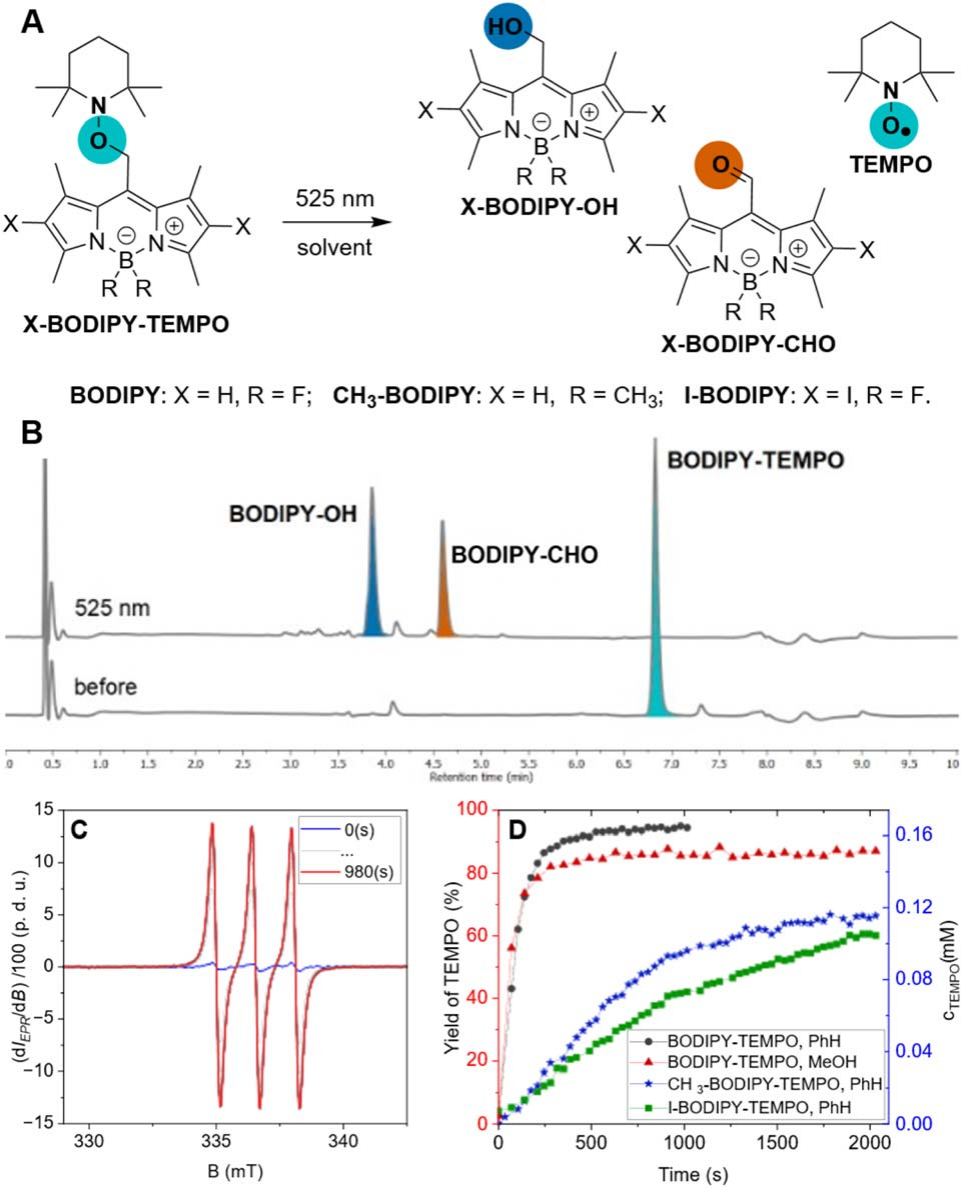
(A) Reactivity of BODIPY–TEMPO upon illumination with 525 nm light in MeOH and corresponding: (B) traces from LC-MS analysis; (C) evolution of EPR spectra [before (blue), in time (grey), and after (red) illumination]. (D) Concentration (blue axis) and yield (red axis) of TEMPO released over time from CH_3_–BODIPY–TEMPO (blue stars, PhH), I–BODIPY–TEMPO (green squares, PhH), BODIPY–TEMPO (black circles, PhH: red triangles, MeOH).

Photoinduced *release* of TEMPO was investigated on three BODIPY scaffolds BODIPY–TEMPO, CH_3_–BODIPY–TEMPO, and I–BODIPY–TEMPO conjugates (Charts 3, S1-2 and S2-3). The spectral properties of the X–BODIPY–TEMPO conjugates closely matched those of the corresponding X–BODIPY–Cl precursors (Tables 2, S2-1, and S2-2), confirming that the TEMPO substituent does not significantly perturb the BODIPY chromophore. Introduction of substituents resulted in decrease in fluorescence quantum yields (*Φ*_fl_) and fluorescence lifetimes (*τ*) and in increase of triplet state quantum yields (*Φ*_Δ_) (Table 2, Fig. S2-5, S2-6, and S2-7). The LC-MS analysis and ^1^H NMR measurements gave evidence for the formation of three products as a result of photorelease from BODIPY–TEMPO: BODIPY–OH, BODIPY–CHO, and TEMPO radical (Charts 3B, S2-18, S2-19, and S2-20). Neither N–O bond cleavage products^8^ nor S_N_1 substitution products were observed, demonstrating that TEMPO *release* proceeds exclusively *via* homolytic bond cleavage. This establishes BODIPY photocages as a scaffold for controlled light-driven radical generation.

**Table 2:** Efficiency of the *release* of TEMPO from X–BODIPY–TEMPO

Code	Solv.	*Φ*_fl_ [%]	*Φ*_Δ_ [%]	*τ*, ns	*Φ*_r_ [%]	Yield [%]
BODIPY–TEMPO	PhH	38	3	5	0.5	95
	MeOH	n.d.	n.d.	n.d.	0.6	85
CH_3_–BODIPY–TEMPO	PhH	15	9	4	0.07	65
I–BODIPY–TEMPO	PhH	2	80	b.d.l.	0.09	62

The photorelease of TEMPO was further supported by the EPR kinetic measurements (Chart 3C and D). Under green light illumination, BODIPY–TEMPO showed high reactivity with 95% of TEMPO released in PhH (Table 2), exceeding the values reported for quinone methide (QM)^18^ and 2-(4-nitrophenyl)benzofuran^17^ photocages (∼80%), and similar to the performance of UV-absorbing pyrene-based photocage (95%).^19^ Our system achieved an excellent uncaging cross-section value (*δ*_r_ ∼390 M^−1^ cm^−1^) and the highest photorelease quantum yield (*Φ*_r_ = 0.5%) reported so far for green-light-induced radical release (compared to the 0.047% for a QM photocage^18^). Contrary to BODIPY–Cl, which underwent partial photo-S_N_1 reaction in MeOH, BODIPY–TEMPO afforded only the products of homolysis with 85% yield in MeOH, demonstrating that the nucleophilic solvent does not affect the photoinduced homolysis. Boron methylation and iodination of the BODIPY-core, however, resulted in a up to ∼7-fold decrease of the photorelease efficiency and a loss of ∼30% in the yield of released TEMPO.

These results are consistent with the reduced radical reactivity observed for I–BODIPY–Cl and CH_3_–BODIPY–Cl in the *catch* experiments. The reduced reactivity may be caused by nitroxyl radicals released during the reaction progress, which might promote the intersystem crossing (ISC) from the singlet and triplet excited states,^26–31^ as demonstrated on a series of BODIPY derivatives.^32–34^ Therefore, TEMPO released during the reaction may influence the photodynamics and efficiency of the system by promoting ISC and other deactivation pathways. Altogether, the photoreactivity of X–BODIPY–Cl/TEMPO demonstrated that the homolytic BODIPY–payload bond cleavage is more efficient for compounds with the high *Φ*_fl_, and therefore could originate from the excited singlet state.

### Polymerization

**Scheme 2:**
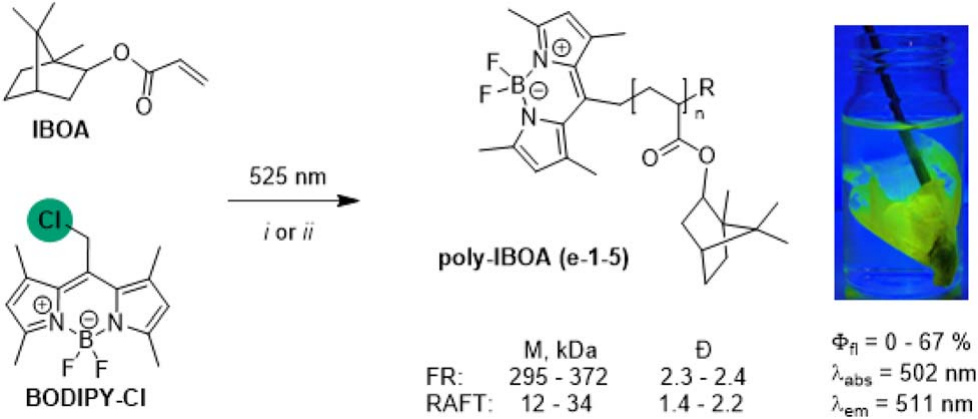
FR (i) and RAFT (ii), DDMAT RAFT agent polymerization of IBOA upon irradiation with 525 nm light using BODIPY–Cl photoinitiator (Table S3-1: e-1-5) and overview of the polymers' properties (labs, *λ*_em_ – absorbance and fluorescence maxima, *Φ*_fl_, *M*_n_ – molecular weight, and *Đ* – dispersity).

Taking advantage of the facile radical reactivity of BODIPY–Cl, we then explored the possibility of using BODIPY–Cl as a Type I radical photoinitiator for the synthesis of fluorescent isobornyl acrylate (IBOA) polymers through a Giese-type radical addition to the activated C–C double bond of IBOA (Scheme 2).

Free radical (FR) polymerization of BODIPY–Cl (0.08 mol%) led to low yields of polyacrylates (poly-IBOA, 11–19%), with high molecular weight (*M*_n_ = 295–372 kDa) and high dispersity (*Đ* ∼2.3, Table S3-1). To improve the dispersity of prepared fluorescent polymers, BODIPY–Cl was further exploited as photoinitiator in a RAFT polymerization, leading to poly-IBOAs of lower molecular weights (*M*_n_ = 11.6–34.1 kDa) and in higher yields (up to 69%) with excellent *Đ* (∼1.5) at the lower initiator concentrations (Fig. S3-9A: e-4c, Table S3-1). Solutions of poly-IBOAs prepared under the same conditions using both FR and RAFT approaches (e-1a,b, Table S3-1) have identical absorption and emission spectra (*λ*_abs_ = 502 nm, *λ*_em_ = 511 nm) with *Φ*_fl_ ∼42%, comparable to the values measured for the standard compound BODIPY–OAc (∼48%, Fig. S3-7) indicating that attaching the BODIPY fluorophore onto a polymer chain does not significantly decrease its brightness. Kinetic studies showed that the conversion rate and properties of the resulting poly-IBOAs (*Φ*_fl_, *M*_n_, *Đ*) can be efficiently controlled by the irradiation time: longer irradiation time led to higher conversions and a decrease in *Φ*_fl_ associated with the dye photobleaching (Fig. S3-6, S3-7, S3–9B: e-5, Table S3-1). While studying the polymerization mechanism, we detected a BODIPY–RAFT intermediate analogous to the BODIPY–TEMPO conjugate (Fig. S3-10), indicating that the *catch-and-release* mechanism extends to RAFT agents and that light-induced radical generation is not limited to the initiation phase.

## Conclusions

In conclusion, we demonstrated that BODIPY photocages can efficiently release radical payloads *via* homolytic cleavage of the photocage–payload bond, challenging the prevailing evidence that BODIPY uncaging proceeds exclusively through heterolytic pathways. This insight enabled us to design a reversible radical *catch-and-release* system based on homolytic bond formation and cleavage, allowing straightforward caging of radical payloads and their efficient photorelease. Using TEMPO as a model payload, radical release was found to be four times more efficient than heterolytic acetate uncaging from the analogous BODIPY–OAc compound. Structure–reactivity analysis of X–BODIPY–TEMPO revealed that radical photorelease is favoured for cages with high fluorescence quantum yields (X = H) and diminishes upon boron methylation (X = CH_3_) or iodination (X = I). As a representative application, we exploited the radical reactivity of BODIPY–Cl in free radical and RAFT polymerizations of isobornyl acrylate, yielding polymers covalently linked to the BODIPY core. Detection of a BODIPY–RAFT intermediate analogous to the BODIPY–TEMPO conjugate confirmed that the *catch-and-release* mechanism participates in the RAFT process beyond the initiation phase. This work establishes that BODIPY photocage reactivity is not intrinsically heterolytic but is governed by payload character, reaction conditions, and photocage photophysics. Optimizing these factors enables predictable light-controlled radical generation under mild conditions, and opens opportunities for late-stage photochemical payload installation.

## Author contributions

Conceptualization: A. P., S. G.-L., T. S. Data curation: A. P., A. V., K. B., J. T. Formal analysis: A. P., A. V., J. T. Funding acquisition: A. P., S. G.-L., T. S. Investigation: A. P., A. V., K. B., J. T. Methodology: A. P., J. T. Project administration: A. P. Resources: A. P. Supervision: A. P., S. G.-L., T. S. Validation: A. P. Visualization: A. P. Writing – original draft: A. P. Writing – reviewing and editing: A. P., A. V., K. B., J. T., S. G.-L., T. S.

## Conflicts of interest

There are no conflicts to declare.

## Supplementary Material

SC-017-D6SC01848C-s001

## Data Availability

The raw data that support the findings of this study are openly available in ZENODO at https://10.5281/zenodo.17405857. A preprint version of this work has been posted on ChemRxiv (https://chemrxiv.org/doi/full/10.26434/chemrxiv-2025-96t6r/v2). Supplementary information (SI): general procedures, synthetic procedures, structure characterization, photophysical and photochemical properties of the BODIPY derivatives, polymerization procedures, and characterization of polymers. See DOI: https://doi.org/10.1039/d6sc01848c.
