# Supplementary material for: Light-driven radical catch-and-release with BODIPY photocages
Source: Chem Sci. 2026 Apr 27;17(23):11430–5. doi: 10.1039/d6sc01848c (PMC13140688; doi:10.1039/d6sc01848c)
Supplement: SC-017-D6SC01848C-s001 [file SC-017-D6SC01848C-s001.pdf]

Supplementary Information (SI) for Chemical Science.
This journal is © The Royal Society of Chemistry 2026

Supporting Information

Light-Driven Radical Catch-and-Release with BODIPY Photocages

Anna Poryvai,*[ab] Anna Vasiļevska,[bc] Karolína Bangievská,[b] Ján Tarábek,[b] Sandrine Gerber-Lemaire[a] and Tomáš Slanina[b]

[a] Institute of Chemical Sciences and Engineering, Ecole Polytechnique Fédérale de Lausanne, EPFL SB ISIC SCI-SB-SG, Station 6, CH-1015 Lausanne, Switzerland

[b] Institute of Organic Chemistry and Biochemistry of the Czech Academy of Sciences Flemingovo náměstí 542/2, Prague 6, 160 00, Czech Republic

[c] Department of Organic Chemistry, Faculty of Science, Charles University, Albertov 6, 128 00 Prague 2

E-mail: anna.poryvai@epfl.ch

Contents

S1. Synthesis and characterization 2

S1.1 General information 2

S1.2 Synthesis of BODIPY derivatives 3

S1.3 NMR and HR-MS spectra 6

S2. Photophysical properties and light-induced reactivity 11

S2.1 Absorption and emission spectra, fluorescence quantum yields 11

S2.2 Photoreactivity of BODIPY-Cl in benzene, methanol, and *tert*-butanol 15

S2.3 Photoreactivity of BODIPY-TEMPO derivatives in benzene, methanol, *t*-BuOH 24

S3. Polymers 29

S4. References 37

S1. Synthesis and characterization

S1.1 General information

Commercially available reagents were purchased from Merck (Germany), Penta Chemicals (Czech Republic), and Fluorochem Ltd. (United Kingdom) and used without further purification. Dry solvents were purchased from Acros Organics (part of Thermo Fisher Scientific, Belgium). Solvents are abbreviated as follows: cyclohexane (CyH), benzene (PhH), dichloromethane (DCM), dimethylsulfoxide (DMSO), ethyl acetate (EtOAc), methanol (MeOH), 2-methylpropan-2-ol (*tert*-butanol, *t*-BuOH), toluene (PhMe). Reaction progress was followed using thin-layer chromatography (TLC) plates (Macherey-Nagel, Germany). Silicagel 40–60 µm (Lach-Ner, Czech Republic) was used for column chromatography. Column chromatography was performed using the ECOM system for flash chromatography. Ratios of solvents are presented in v/v.

Purity of the isolated compounds was assessed using an LC-MS-2020 system from Shimadzu Corporation (Japan) with UV-VIS and ESI-MS detectors on a CORTECS C18+ RP-column (2.7 µm, 4.6 × 50 mm) equipped with a VanGuard (3.9 × 5 mm) pre-column from Waters (USA). Compounds were eluted with a mixture of acetonitrile and water with formic acid (0.1 % *v/v*) in a gradient mode, starting with 95 % and ending with 5 % of water. HPLC-grade acetonitrile from Fisher Scientific (UK), LC-MS grade formic acid from VWR (Belgium), and Milli-Q water were used for chromatography.

The structure of products was confirmed by NMR spectroscopy and mass spectrometry. ^{1}H and ^{13}C NMR spectra were recorded on a Bruker Avance III™ HD 400 MHz spectrometer at 400.13 and 100.60 MHz, respectively. Solvents used for measurements are specified for each sample. The residual solvent signals were used as a reference. Chemical shifts (δ) are given in ppm, spin-spin coupling constants (J) in Hz. The multiplicity of signals is described as follows: singlet (s), broad singlet (br s), doublet (d), triplet (t), quartet (q), multiplet (m). High-resolution ESI mass spectra (HRMS) were recorded using an LTQ Orbitrap XL (Thermo Fisher Scientific, USA) hybrid FT mass spectrometer with a linear ion trap MS and the Orbitrap mass analyzer. IR spectra were recorded using an FT-IR Frontier (Perkin Elmer Inc) spectrometer equipped with an ATR setup (Quest ATR Diamond, Specac Ltd). The band wavenumbers are given in cm^{-1}, and their intensity is provided as follows: very strong (vs), strong (s), medium (m), weak (w), broad (br).

Absorption spectra of target compounds were recorded using an Agilent Cary 8454 UV-Vis spectrometer. Emission spectra were recorded using a Horiba Duetta spectrometer. All spectra were measured in cells for fluorescence (3.0 mL volume; 10 mm optical path). Custom-made LED light source with the maximum wavelength at 525 nm (Fig. S2-23, $F_{(525)} = 4.2 \times 10^{-7}$ Einstein s^{-1})[1] was used for kinetic measurements. The LED source was placed in a custom-made holder, ensuring identical orientation of LEDs around the sample and perpendicular irradiation of the sample with respect to the beam used for recording UV-VIS spectra. The emission spectrum of LED source is presented in Fig. S2-23.

The EPR spectra of the reactivity of **X-BODIPY-Cl** were acquired on Bruker EMXplus 10/12 CW (continuous wave) spectrometer equipped with a Premium-X-band microwave bridge and the standard rectangular probehead/cavity (ER160FCQ, Bruker). The g_{iso} value of radicals was determined using a built-in spectrometer frequency counter and a ER036TM NMR-Teslameter (Bruker). Typical uncertainty for the g-value determination reads ±0.0002. Irradiation was done

by home-made green (525 nm) small LED-array, which irradiated sample through the grid of the above-described rectangular probehead. In order to effectively irradiate the largest area, samples were injected into a special EPR flat-cell (ER165FCVT-Q, Bruker). Prior to each irradiation, a blank experiment by LED-off-state was performed in order to exclude any EPR signal intensity increase/decline (based on the reaction performed, vide infra) before light-treatment. Spin trapping experiments were acquired by using the PBN (*N*-*tert*-Butyl-α-phenylnitrone) as a spin trap agent. This was supplied by Sigma-Aldrich (for ESR spectroscopy, crystallic, assay ≥99.5 % HPLC). Because of low EPR signal-to-noise ratio, which did not enable direct integration of EPR spectra, each EPR spectrum (within the time series) had to be simulated individually and finally, the double integration was performed on simulated spectra. The entire procedure was done within the Xenon (Bruker) acquisition/processing software (Bruker 2025).[3] The kinetic measurements on **X-BODIPY-TEMPO** compounds were done in the glass capillaries (1 mm i.d.) using a benchtop setup, Magnettech ESR5000 from Bruker, equipped with a 525 nm laser.

Size exclusion chromatography was performed on an Agilent 1260 Infinity chromatograph equipped with a 390-MDS refractive index detector and Mistral column oven using precolumn PSS SDV and 2 x column PSS SDV analytical linear XL at the following conditions: eluent – THF, flow rate – 1 mL/min, temperature – 40 °C. The calibration curve was measured using conventional polystyrene narrow standards ranging from 682 – 2 520 000 g/mol. The MW calculations were performed with Cirrus software.

S1.2 Synthesis of BODIPY derivatives

The synthetic route to the target materials is depicted in Scheme S1-1. The starting **BODIPY-Cl,** its iodinated (**I-BODIPY-Cl**) and methylated (**CH_3-BODIPY-Cl**) derivatives were prepared according to the literature.[2]

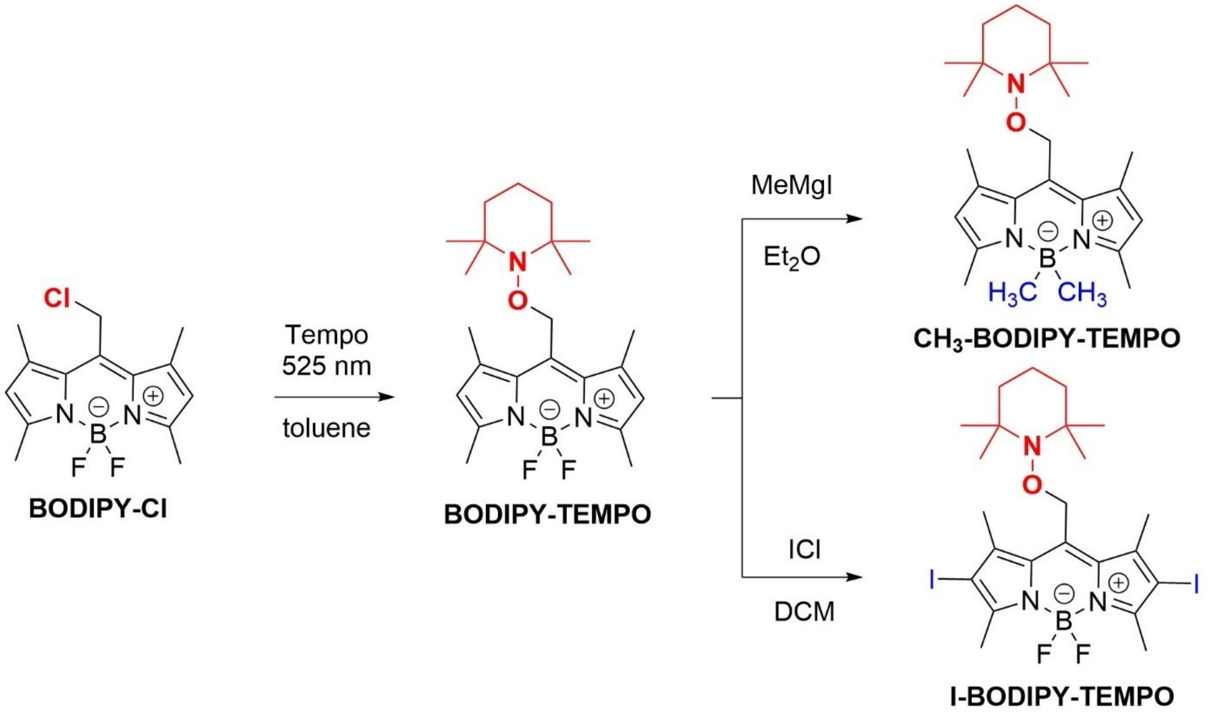

Scheme S1-1: Synthesis of BODIPY-TEMPO derivatives

BODIPY-TEMPO

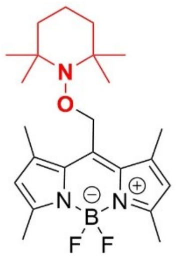

A solution of BODIPY-Cl (22 mg, 0.07 mmol, 1 eq.) and TEMPO (125 mg, 0.8 mmol, 11 eq.) in PhMe (20 mL) was pumped through the PTFE capillary (internal diameter 0.75 mm, length 3.5 m, flow rate 0.05 mL/min) irradiated with the green light LED (525 nm). The solvent was removed under reduced pressure, and the crude mixture was purified using column chromatography (eluent: gradient from CyH to CyH/DCM/EtOAc, 9/1/1), yielding **BODIPY-TEMPO** as a pink crystalline product (27 mg, 87 %).

^{1}H NMR (400 MHz, $CDCl_3$): δ 6.05 (s, 2H), 5.20 (s, 2H), 2.55 (s, 6H), 2.52 (s, 6H), 1.43 (m, 4H), 1.20 (m, 2H), 1.07 (s, 6H), 1.04 (s, 6H).

^{13}C NMR (101 MHz, $CDCl_3$): δ 155.2 (C), 142.0 (C), 139.2 (C), 132.5 (C), 122.0 (CH), 70.4 (CH_2), 59.9 (C), 39.9 (CH_2), 33.4 (CH_3), 20.7 (CH_3), 17.4 (CH_2), 16.6 (CH_3), 14.8 (CH_3).

HR-MS (ESI+), *m/z*: $[M+H]^+$, calcd for $C_{23}H_{35}ON_3BF_2^+$ 418.28358, found 418.28381.

CH_3-BODIPY-TEMPO

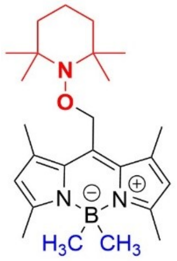

A solution of MeMgI (3.0 M in diethyl ether, 0.1 mL, 0.3 mmol, 5.0 eq.) was added to a solution of BODIPY-TEMPO (27 mg, 0.06 mmol, 1.0 eq.) in dry Et_2O (5 mL) at 0°C. The reaction mixture was stirred at 0°C for 30 min and warmed up to r. t. Water (5 mL) was added, and the product was extracted with DCM. The product was purified by column chromatography (eluent: CyH/DCM/EtOAc, 9/1/1) to afford **CH_3-BODIPY-TEMPO** as red crystals (17 mg, 64%).

^{1}H NMR (400 MHz, DCM-*d2*) δ 6.07 (s, 2H), 5.28 (s, 2H), 2.57 (s, 6H), 2.44 (s, 6H), 1.53 (m, 4H), 1.30 (m, 2H), 1.04 (m, 12H), 0.14 (s, 6H).

^{13}C NMR (101 MHz, DCM-*d2*): δ 152.2 (C), 140.1 (C), 138.5 (C), 131.6 (C), 122.7 (CH), 71.1 (CH_2), 60.2 (C), 40.3 (CH_2), 33.5 (CH_3), 30.3 (CH_2), 20.9 (CH_3), 17.2 (CH_3), 17.0 (CH_3), 9.9 (CH_3).

HR-MS (ESI+), *m/z*: $[M+H]^+$, calcd for $C_{25}H_{41}BN_3O^+$ 410.33372, found 410.33414.

I-BODIPY-TEMPO

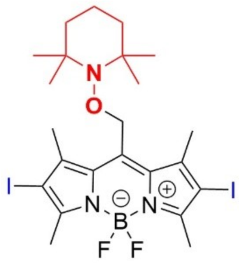

To a solution of **BODIPY-TEMPO** (43 mg, 0.1 mmol, 1.0 eq.) in dry DCM (2 mL), a solution of ICl (37 mg, 0.2 mmol, 2.2 eq) in DCM (1 mL) was added dropwise at 0 °C. The reaction mixture was stirred at 0°C for 1 h and warmed up to r.t. The solution was transferred onto a prep-TLC plate. After DCM evaporation (r.t., in dark), the target product was eluted using CyH/EtOAc/DCM, 9/1/1, yielding I-BODIPY-TEMPO as pink crystals (15 mg, 22%).

^{1}H NMR (400 MHz, DCM-*d2*): δ 5.29 (s, 2H), 2.62 (s, 6H), 2.59 (s, 6H), 1.41 (m, 4H), 1.28 (d, *J* = 11.0 Hz, 2H), 1.05 (s, 6H), 1.04 (s, 6H).

^{13}C NMR (101 MHz, DCM-*d2*): δ 156.9 (C), 144.6 (C), 139.6 (C), 132.6 (C), 87.3 (C), 71.1 (CH_2), 60.4 (C), 40.2 (CH_2), 33.5 (CH_3), 30.2, 20.9 (CH_3), 19.5 (CH_3), 17.7. (CH_2), 16.6 (CH_3).

HR-MS (ESI+), *m/z*: $[M+H]^+$, calcd for $C_{23}H_{33}BF_2I_2N_3O^+$ 670.07686, found 670.07764.

S1.3 NMR and HR-MS spectra

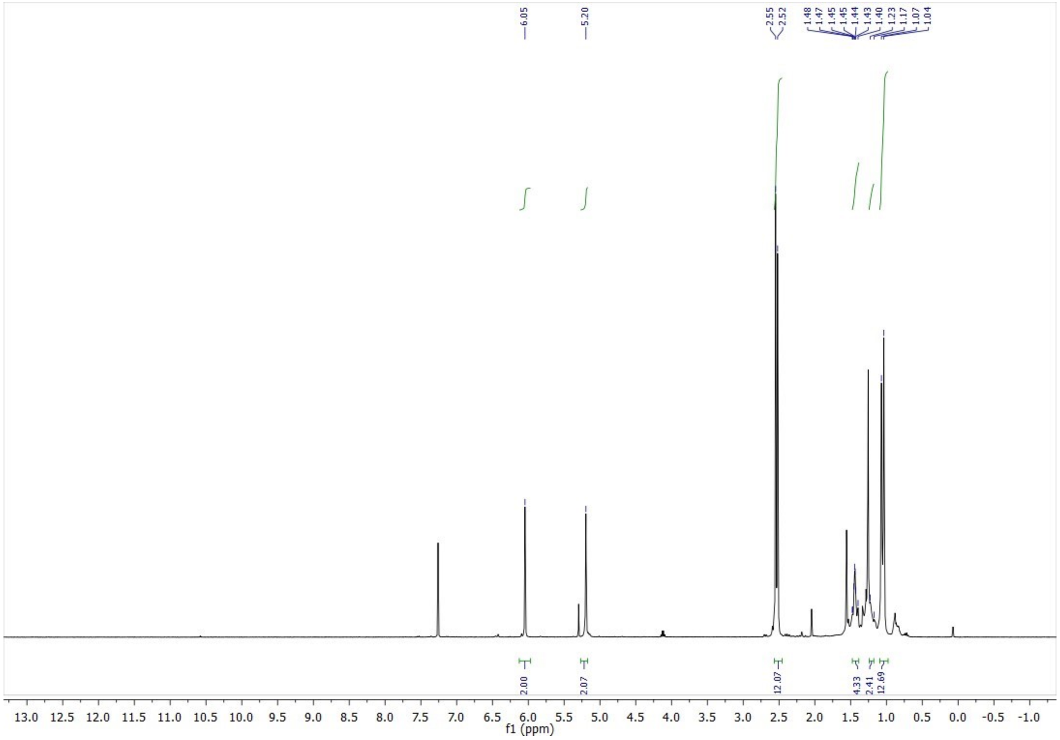

Figure S1-1: ^{1}H NMR spectrum of **BODIPY-TEMPO** in $CDCl_3$

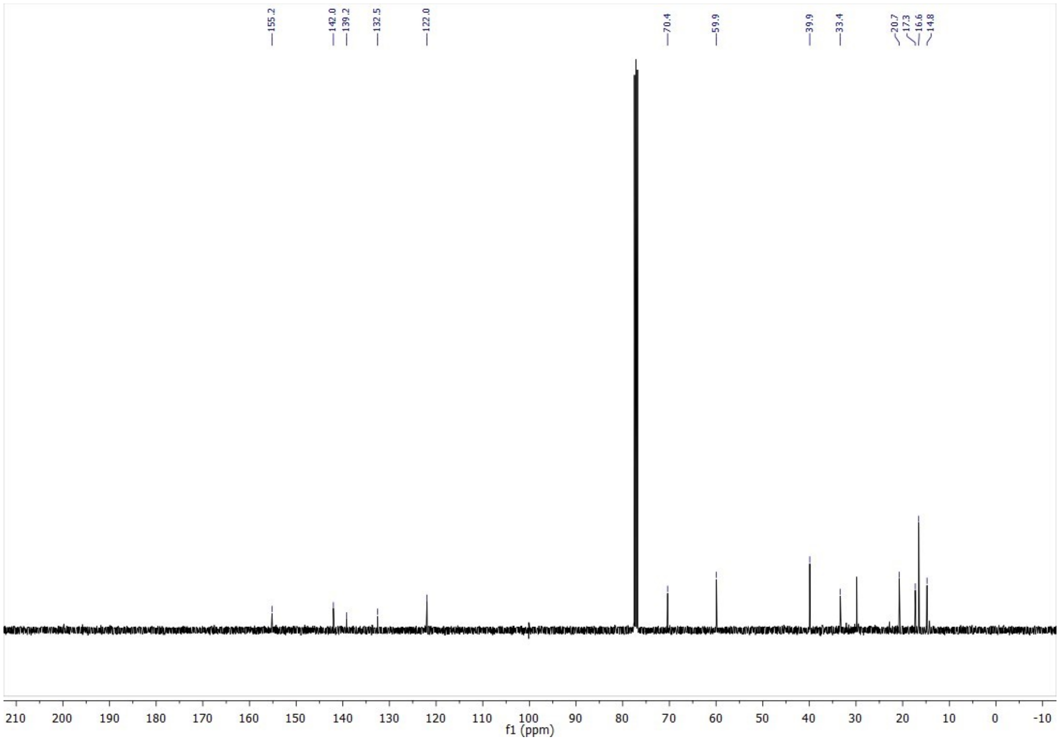

Figure S1-2: ^{13}C NMR spectrum of **BODIPY-TEMPO** in $CDCl_3$

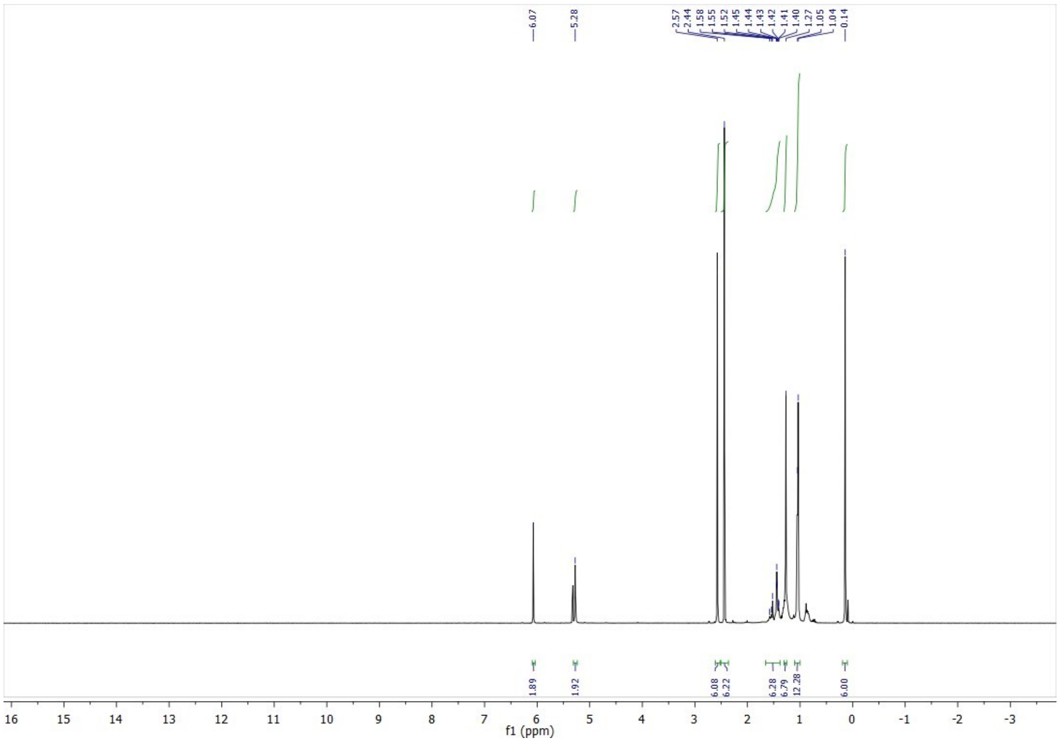

Figure S1-3: ^{1}H NMR spectrum of **CH_3-BODIPY-TEMPO** in DCM-*d2*

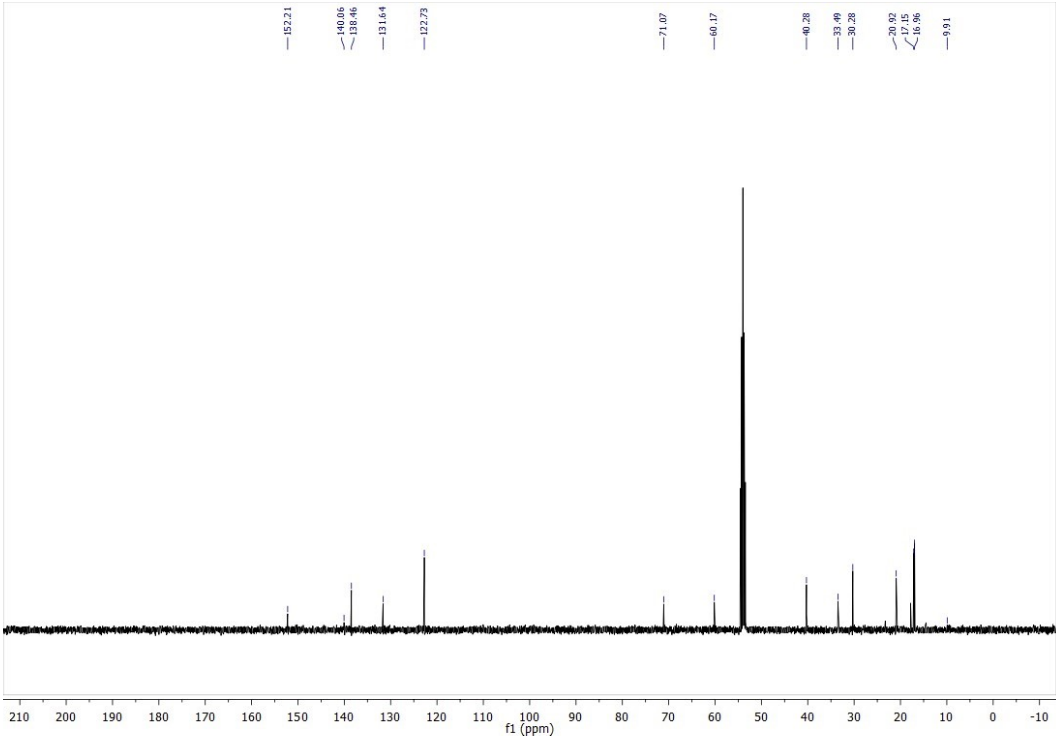

Figure 1-4: ^{13}C APT NMR spectrum of **CH_3-BODIPY-TEMPO** in DCM-*d2*

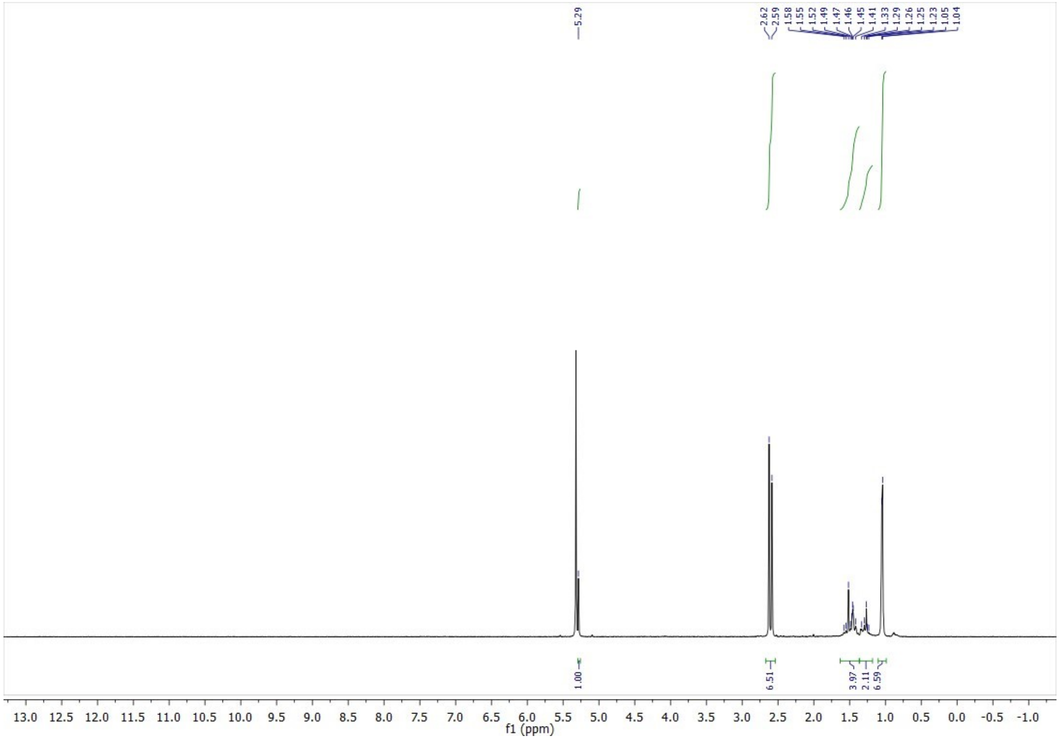

Figure S1-5: ^{1}H NMR spectrum of **I-BODIPY-TEMPO** in DCM-*d2*

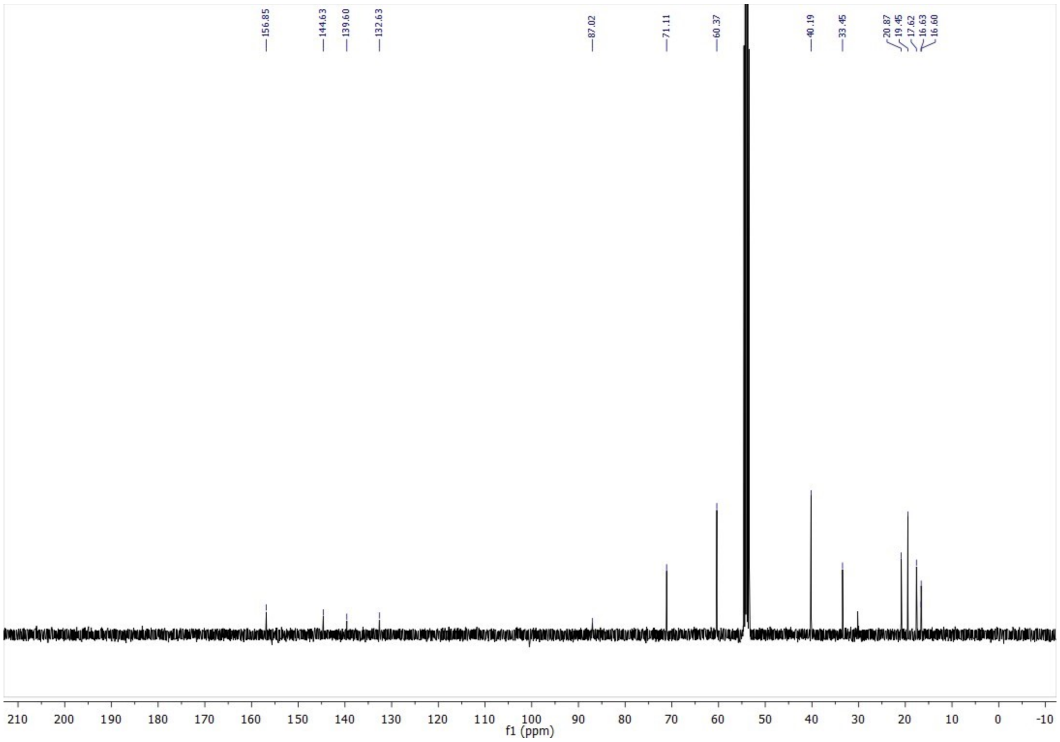

Figure S1-6: ^{13}C NMR spectrum of **I-BODIPY-TEMPO** in DCM-*d2*

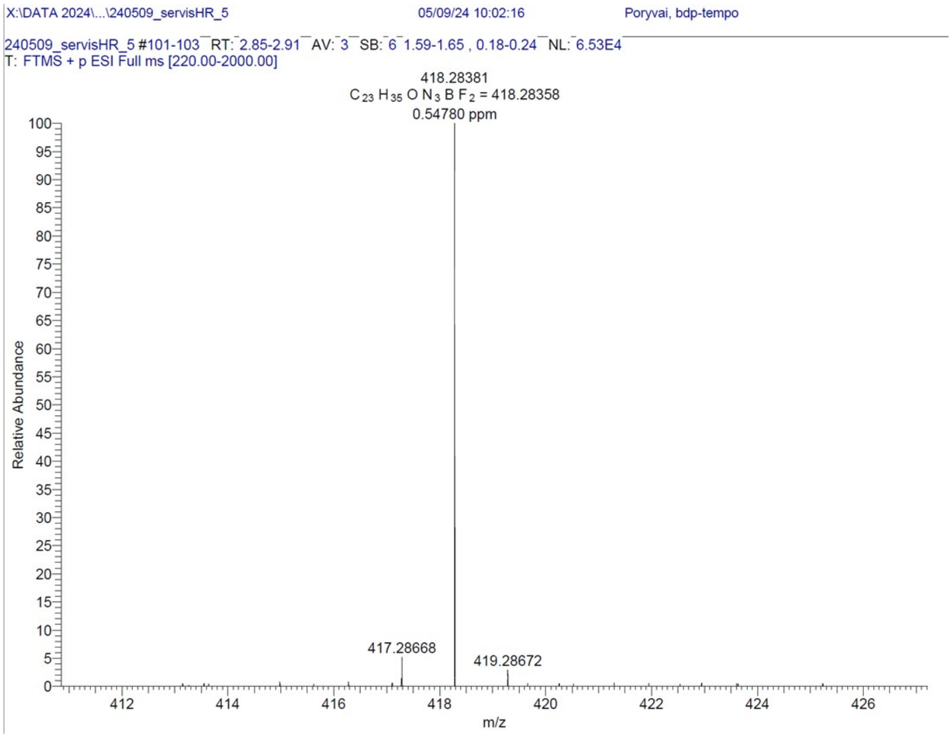

Figure S1-7: HR-MS spectrum of **BODIPY-TEMPO**

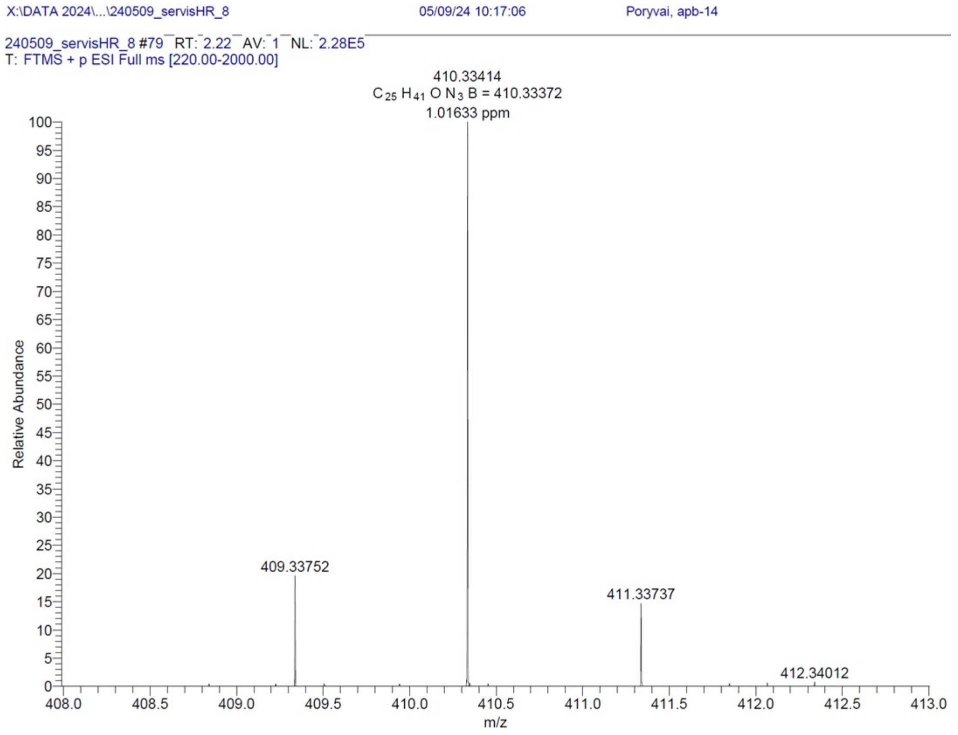

Figure S1-8: HR-MS spectrum of **CH_3-BODIPY-TEMPO**

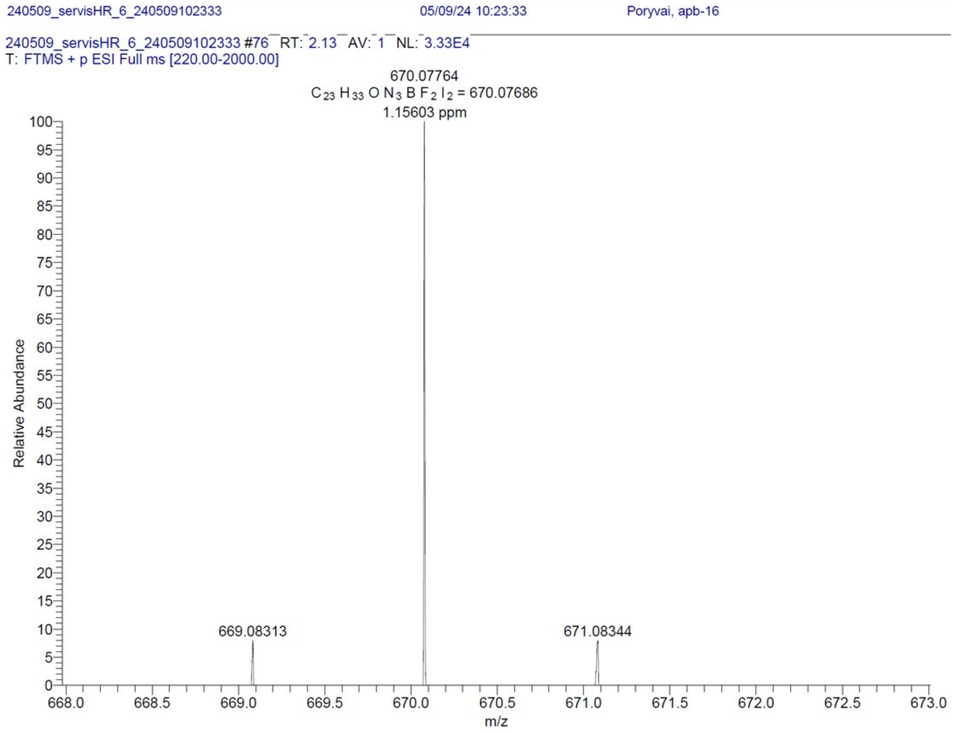

Figure S1-9: HR-MS spectrum of **I-BODIPY-TEMPO**

S2. Photophysical properties and light-induced reactivity

S2.1 Absorption and emission spectra, fluorescence quantum yields

The studied **X-BODIPY-Cl** compounds are known from the literature. Their photophysical properties (absorption (abs) and emission (em) maxima λ_{max} (nm), molar extinction coefficients ε ($M^{-1}cm^{-1}$) at $\lambda_{max,abs}$) in PhH and MeOH are summarized in Table S2-1. Values taken from the literature are marked with the corresponding citation.[2]

Table S2-1: Absorption and emission properties of **BODIPY-Cl**, **CH_3-BODIPY-Cl**, **I-BODIPY-Cl** in MeOH and in PhH.

	$\lambda_{max,abs}$ [nm]		ε [$M^{-1}cm^{-1}$]		$\lambda_{max,em}$ [nm]
	MeOH	PhH	MeOH	PhH	MeOH
**BODIPY-Cl**	523	531	81 870	107 440	534
**CH_3-BODIPY-Cl**	520	526	60 580[28]	62 700	556[28]
**I-BODIPY-Cl**	557	565	59 300[28]	60 000	574[28]

Photophysical properties and quantum yields of fluorescence of **X-BODIPY-TEMPO** and **X-BODIPY-Cl** compounds are summarized in Table S2-2.

Table S2-2: Photophysical properties of **X-BODIPY-TEMPO**, in PhH and of **X-BODIPY-Cl** in PhH and MeOH.

	Solv.	$\lambda_{max,abs}$ [nm]	ε [$M^{-1}cm^{-1}$]	$\lambda_{max,em}$ [nm]	Φ_{fl} [%]
**BODIPY-TEMPO**	PhH	519	78 225	538	38
**CH_3-BODIPY-TEMPO**	PhH	515	98 900	540	15
**I-BODIPY-TEMPO**	PhH	552	65 250	575	2
**BODIPY-Cl**	PhH	531	107 440	545	34
**BODIPY-Cl**	MeOH	523	81 870	534	14
**CH_3-BODIPY-Cl**	MeOH	526	62 700[28]	556[28]	n.d.
**I-BODIPY-Cl**	MeOH	557	59 300[28]	574[28]	1[28]

Absorption and emission spectra were measured in PhH and MeOH. The emission spectra were measured at the following conditions:

- For **BODIPY-TEMPO**, **CH_3-BODIPY-TEMPO** and **BODIPY-Cl** (as a standard): excitation with 450 nm light, acquisition time 0.05 s;
- **I-BODIPY-TEMPO** and **BODIPY-Cl** (as a standard): excitation with 515 nm light, acquisition time 10 s.

BODIPY-TEMPO

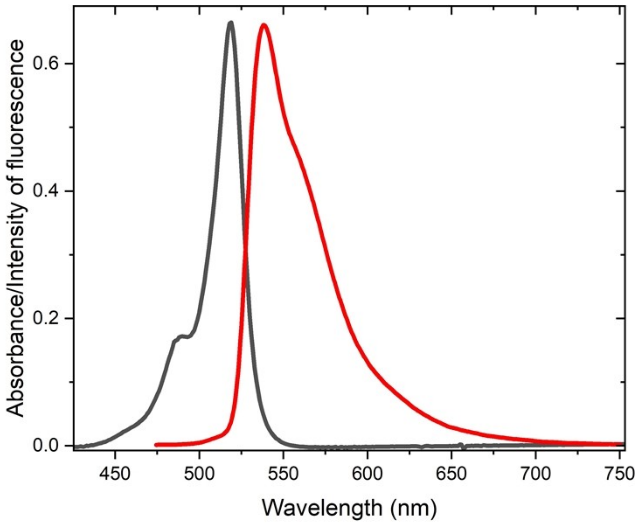

Figure S2-1 Absorption (black) and normalized intensity of fluorescence (red) spectra of **BODIPY-TEMPO** (*c* ~ 8.5 µM) in PhH.

CH_3-BODIPY-TEMPO

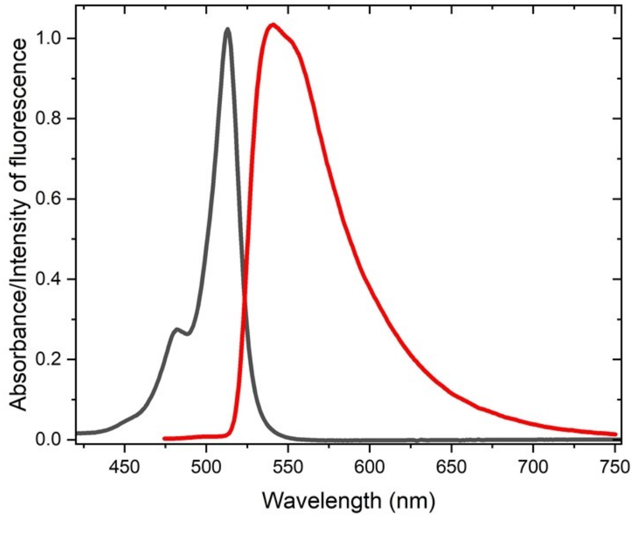

Figure S2-2:Absorption (black) and normalized intensity of fluorescence (red) spectra of **CH_3-BODIPY-TEMPO** (*c* ~ 10 µM) in PhH.

I-BODIPY-TEMPO

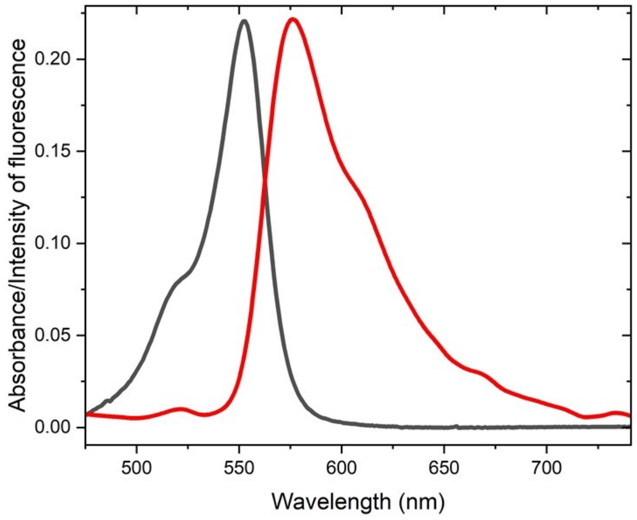

Figure S2-3: Absorption (black) and normalized intensity of fluorescence (red) spectra of **I-BODIPY-TEMPO** (c ~ 3.4 μM) in PhH.

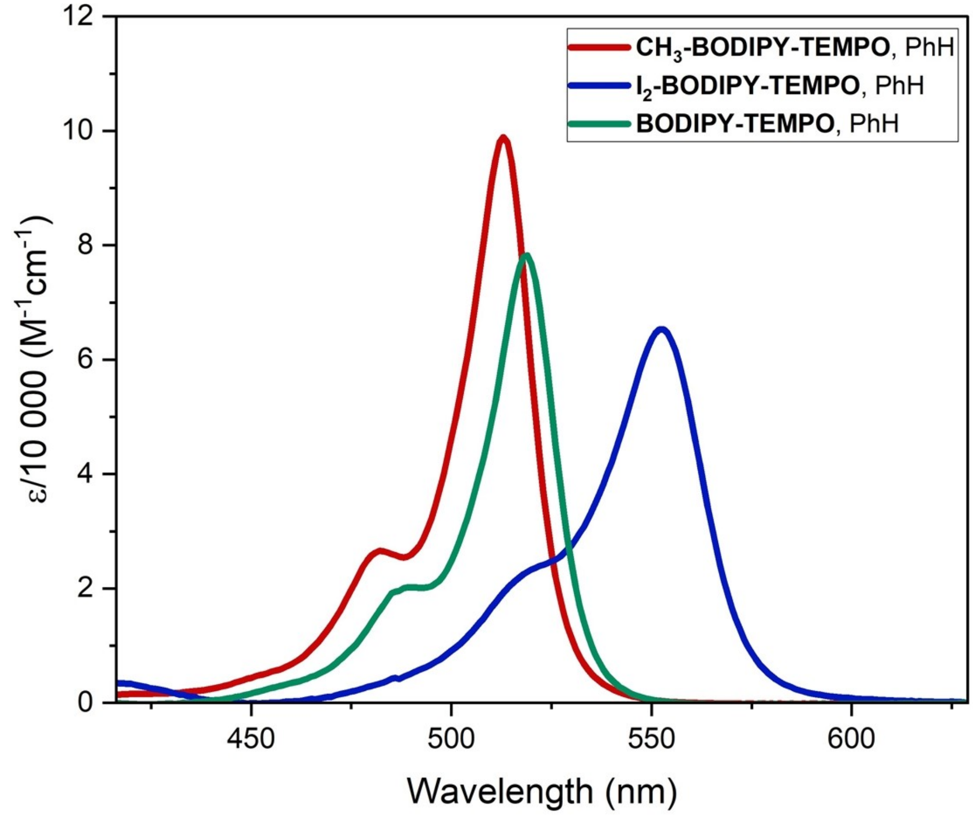

Figure S2-4: Molar extinction coefficients ε ($M^{-1}cm^{-1}$) of **BODIPY-TEMPO** (green), **CH_3-BODIPY-TEMPO** (red), **I-BODIPY-TEMPO** (blue) in PhH.

Quantum yields of fluorescence (Φ_{fl}) of target compounds were determined from a series of absorption and emission spectra at various concentrations of the studied materials. Quantum yields were calculated from the equation below.[4]

$$\Phi_x = \Phi_{st}\left(\frac{Grad_x}{Grad_{st}}\right)\left(\frac{\eta_x^2}{\eta_{st}^2}\right)$$

Φ_x and Φ_{st} are the quantum yields of fluorescence of studied (x) and standard (st) compounds, respectively; η_x^2 and η_{st}^2 are refractive indexes of solvents used for measurements,[5] $Grad_x$ and $Grad_{st}$ are slopes of emission peak areas plotted *vs* values of (1-T), where T stands for transmittance at 450 or 515 nm.

The integrated fluorescence intensities of solutions of **BODIPY-TEMPO**, **CH_3-BODIPY-TEMPO**, **I-BODIPY-TEMPO** and **BODIPY-Cl** plotted *vs.* (1-T) at 450 nm or 515 nm (Fig. S2-5).

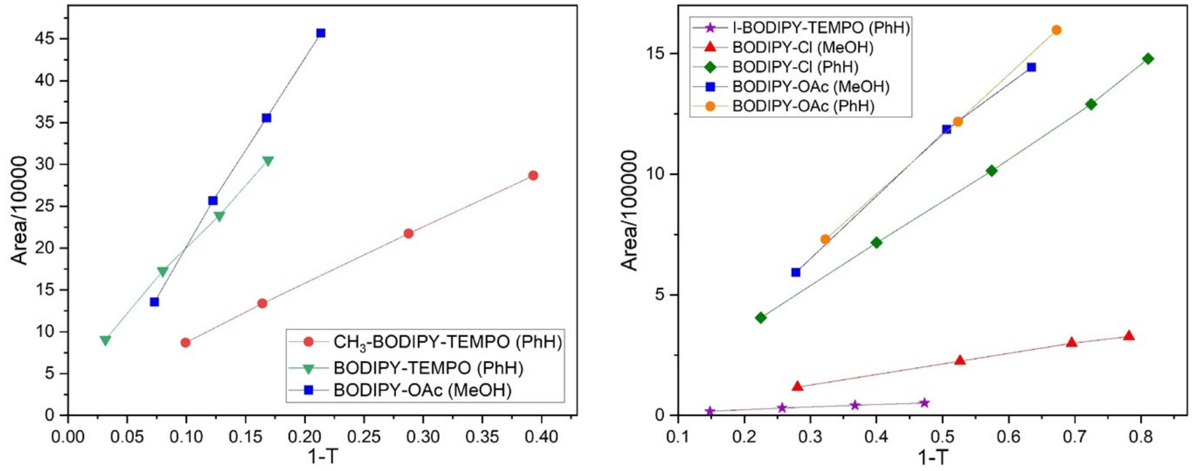

Figure S2-5: Plots for determination of Φ_{fl} for the standard compound **BODIPY-OAc** (MeOH: blue squares, PhH: orange circles), and **BODIPY-Cl** (MeOH: red triangles, PhH: green rhombuses), **BODIPY-TEMPO** (PhH: green triangles), **CH_3-BODIPY-TEMPO** (PhH: red circles), **I-BODIPY-TEMPO** (PhH: violet stars).

The fluorescence lifetime decays (Fig. S2-6) of BOPIDY solutions in benzene (with matched absorbance ~0.2) were recorded on Horiba Fluoromax-4 with 452 nm or 368 nm light source. Obtained decay data were fitted using 1st or 2nd exponential equation (in case of 2nd exponential, the longest half-lifetime value was reported)

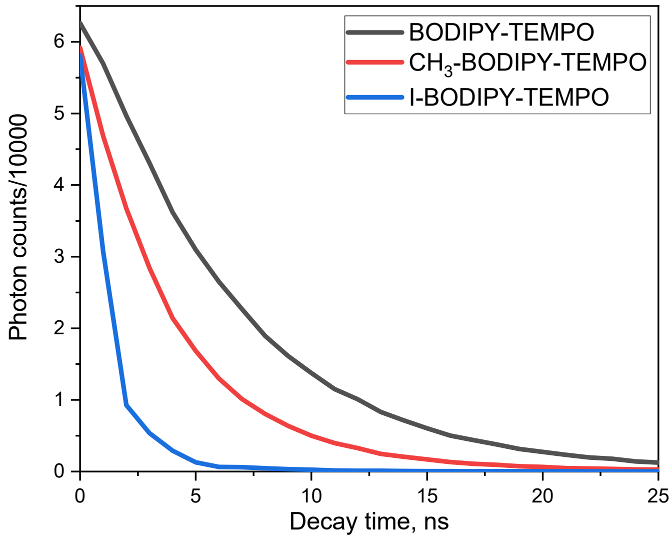

Figure S2-6: The fluorescence lifetime decays of solutions of **BODIPY-TEMPO** (black), **CH_3-BODIPY-TEMPO** (red), **I-BODIPY-TEMPO** (blue) in benzene .

The quantum yields of singlet oxygen production (Φ_Δ) were determined by a published method by monitoring the degradation of 1,3-diphenylisobenzofuran (DPBF) at 410 nm upon photooxidation by generated singlet oxygen (Fig.2-7). Quantum yields were determined by irradiating the solutions (5% acetonitrile in benzene, 2.0 ml, saturated with air) of a photosensitizer (BODIPY dyes or methylene blue (MB), c = 1 $\times 10^{-5}$ M) and DPBF (c ~ 1×10^{-4} M) in a matched 1.0 cm quartz PTFE screw-cap fluorescence cuvette equipped with a stir bar by an LED light source (λ_{max} = 525 nm) and monitoring the initial rate of decrease in the absorption of DPBF. The blank experiment in the absence of photosensitizer did not result in any significant decrease in the absorbance at 410 nm. The quantum yields of singlet oxygen generation (Φ_Δ) were calculated using a relative method according to the equation:

$$\Phi_\Delta^{BODIPY} = \Phi_\Delta^{MB} \frac{k_{BOPIPY}(1 - 10^{10 - A_{MB}})}{k_{MB}(1 - 10^{10 - A_{BODIPY}})}$$

Φ_Δ is the quantum yield of singlet oxygen production (Φ_Δ^{MB} = 0.51),[6] k is the initial rate constant of the decrease in the absorbance of DPBF at 410 nm, and A is the absorbance of the measured solution at the irradiation wavelength (525 nm). Φ_Δ was reported as an average of 3 measurements. The final values of Φ_Δ were corrected for the parallel photorelease process.

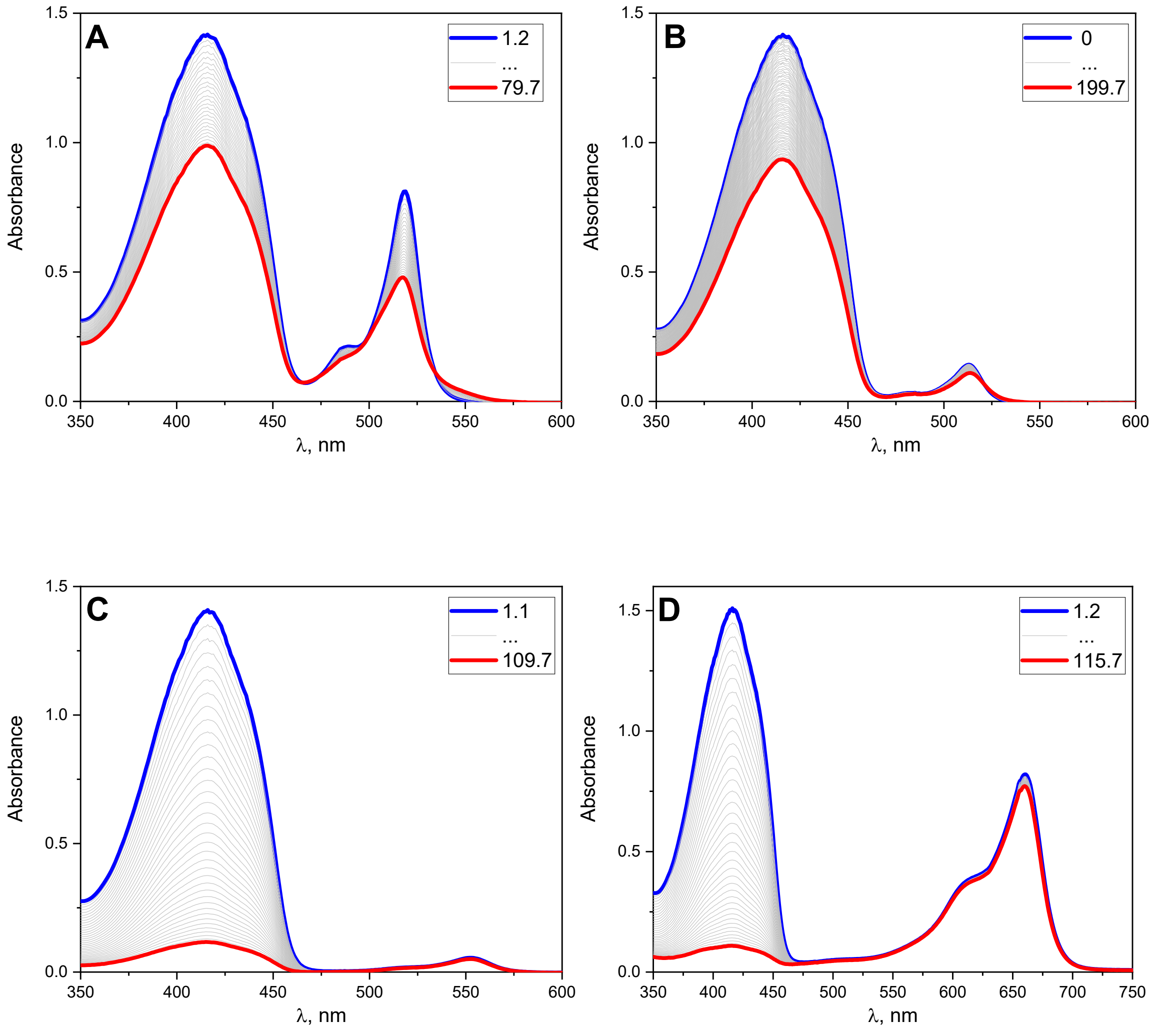

Figure S2-7: Plots for determination of Φ_Δ for **BODIPY-TEMPO** (A), **CH_3-BODIPY-TEMPO** (B), **I-BODIPY-TEMPO** (C), and the standard compound methylene blue (D) in benzene: before (blue, t = 0), over time (grey), and after (red) irradiation (525 nm).

S2.2 Photoreactivity of BODIPY-Cl in benzene, methanol, and *tert*-butanol

The reactivity of **BODIPY-Cl** in various solvents (MeOH, PhH, *t*-BuOH) was studied using UV-Vis, EPR spectroscopies, and LC-MS. The scope of the detected products is summarized in Table S2-3.

Table S2-3: Reactivity of **BODIPY-Cl** with TEMPO at various conditions (-/+, not-/observed in the reaction mixture).

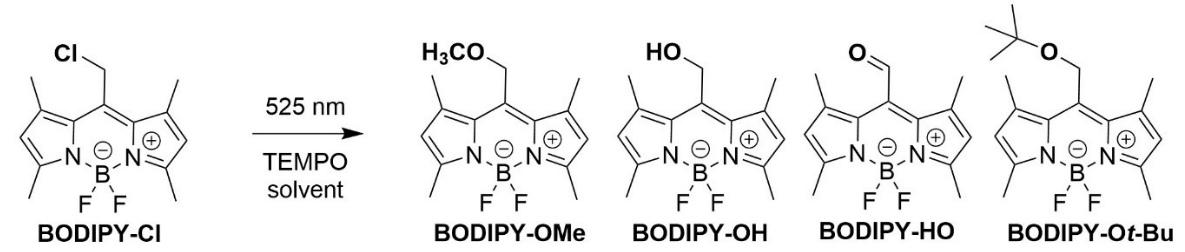

Entry	BODIPY-Cl [M]	TEMPO [M]	MeOH [M]	Solv.	BODIPY-OMe	BODIPY-OH	BODIPY-CHO	BODIPY-O*t*-Bu	t[a] [s]
**A**	$4.5\ 10^{-5}$	-	24.7	MeOH	+	+	-	-	540
**B**	$4.5\ 10^{-5}$	$3.5\ 10^{-4}$	24.7	MeOH	+	+	+	-	480
**C**	$4.5\ 10^{-5}$	-	-	*t*-BuOH	-	+	+	+	1170
**D**	$4.5\ 10^{-5}$	$3.5\ 10^{-4}$	-	*t*-BuOH	-	+	+	+	1170
**E**	$4.5\ 10^{-5}$	-	-	PhH	-	+	-	-	480
**F**	$4.5\ 10^{-5}$	$3.5\ 10^{-4}$	-	PhH	-	+	-	-	480
**G**	$4.5\ 10^{-5}$	-	12.4	PhH	+	+	-	-	480
**H**	$4.5\ 10^{-5}$	$3.5\ 10^{-4}$	12.4	PhH	+	+	+	-	480
**I**	$4.5\ 10^{-5}$	$3.5\ 10^{-4}$	$8.2\ 10^{-3}$	PhH	-	+	-	-	870

[a]: irradiation time

Absorption spectra of the irradiated solutions of **BODIPY-Cl** in various solvents (MeOH, PhOH/MeOH, 1/1, PhH, *t*-BuOH) with or without MeOH or TEMPO were recorded over time upon simultaneous irradiation with 525 nm light (Fig. S2-6). Chromatograms of reaction mixtures before and after irradiation are shown in Fig. S2-7, S2-8.

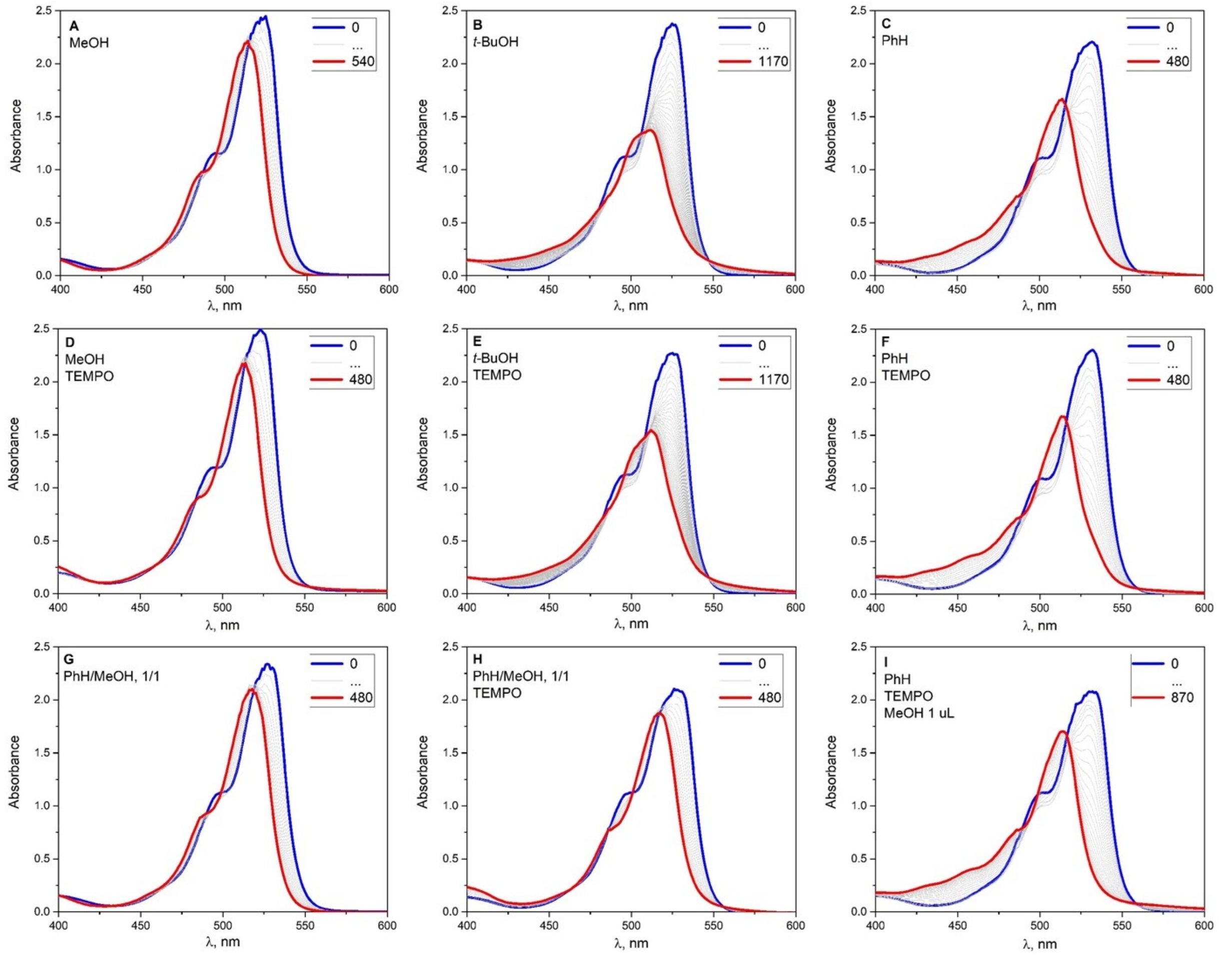

Figure S2-8: Absorption spectra of **BODIPY-Cl** (*c* ~ 45 μM) in MeOH, PhH, PhH/MeOH, *t*-BuOH with/without TEMPO (8 eq.) before (blue, t = 0), over time (grey), and after (t = 480 s) (red) irradiation (525 nm).

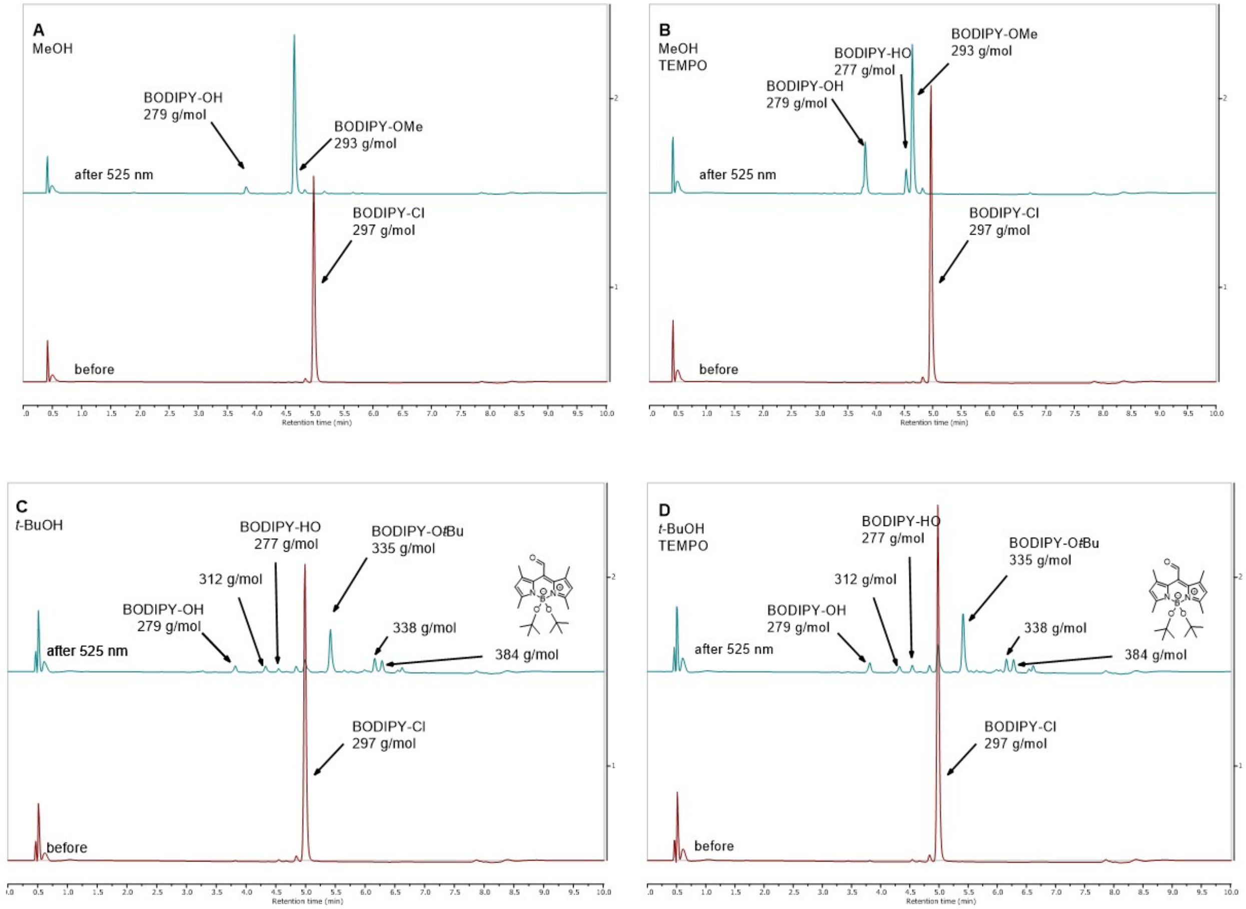

Figure S2-9: Chromatograms from UV-VIS detector (at 450 - 550 nm) of **BODIPY-Cl** with (right) or without (left) TEMPO in MeOH (top) and *t*-BuOH (bottom). Assignment of the compounds is based on the recorded mass spectra (ESI ionization) before (red) and after (blue) irradiation with 525 nm light. A: in MeOH, B: with TEMPO in MeOH, C: *t*-BuOH, D: with TEMPO in *t*-BuOH.

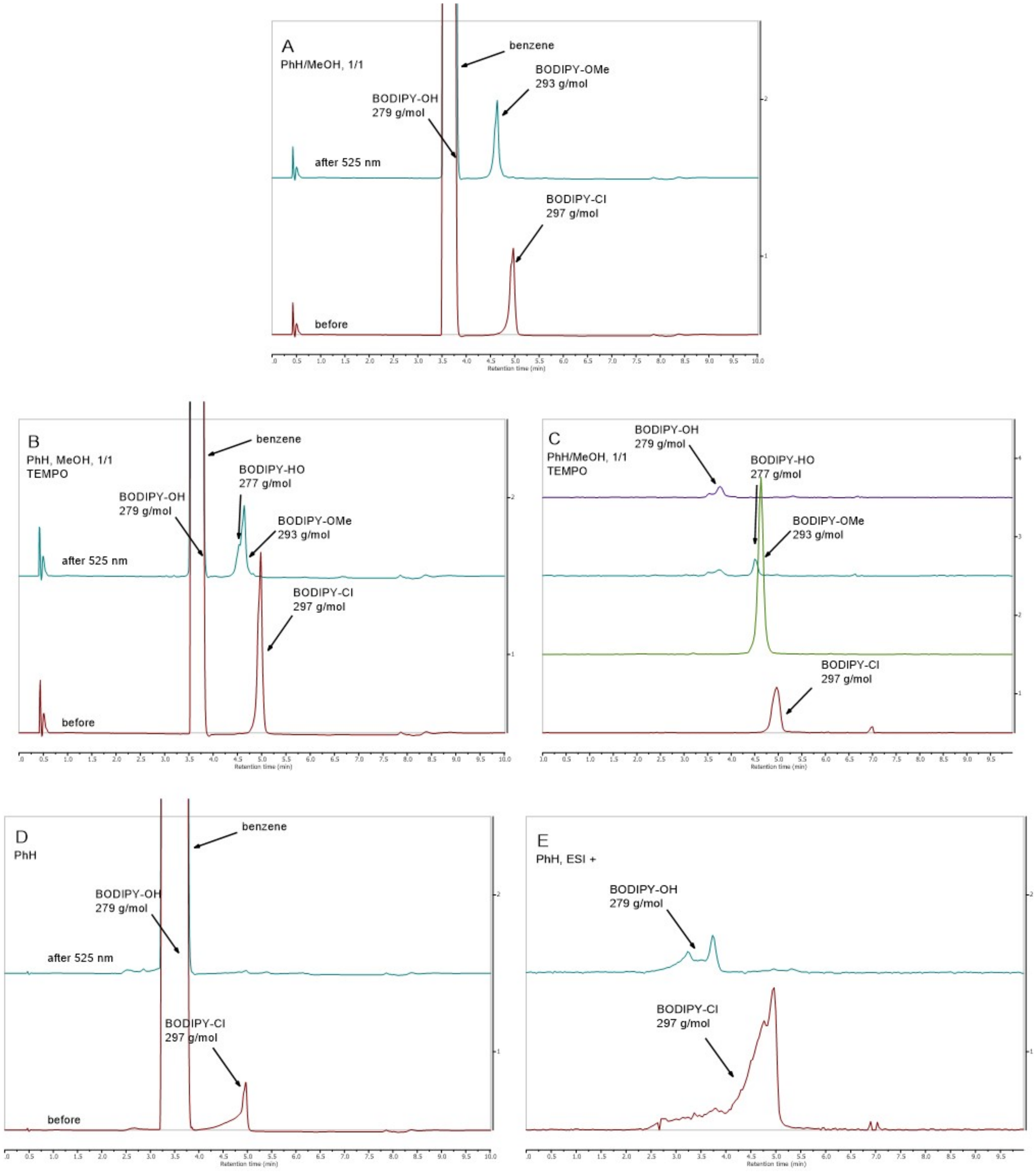

Figure S2-10: Chromatograms from UV-VIS detector at 525 nm (left) of solutions of **BODIPY-Cl** in PhH/MeOH, 1/1 without TEMPO (A) and with TEMPO (B) or in PhH (D) before and after irradiation with 525 nm light. Assignment of the compounds is based on the recorded mass spectra, chromatograms from the ESI detector of the observed ions in the reaction mixtures B and D are shown in panels C and E.

EPR spectra were recorded for solutions of **BODIPY-Cl**, irradiated with 525 nm light in the presence of *N-tert*-butyl-α-phenylnitrone spin-trap (Fig. S2-9) or TEMPO (Fig. S2-10). The spectra gave evidence for the homolytic C-Cl bond cleavage with further trapping of the formed BODIPY radical by *N-tert*-butyl-α-phenylnitrone or TEMPO.

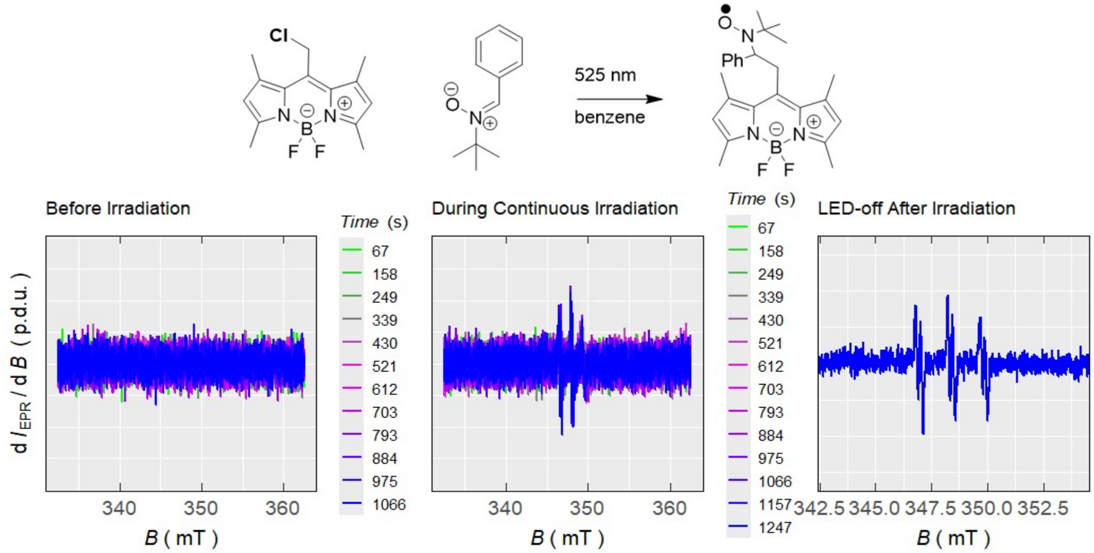

Figure S2-11: EPR responses coming from spin trapping experiment: **BODIPY-Cl** (c = 20 mM in PhH) in the presence of PBN spin trapping agent (c = 25 mM) during the conditioning in the dark (left) and during the continuous irradiation by the 525 nm LED (middle). In addition, an EPR spectrum recorded at LED-off state just after the continuous irradiation (right), showing the stability of the transient radical + PBN adduct. EPR signal detected at $g_{iso} = 2.0054$ with the following hyperfine splitting constants: a($1\times{}^{14}N$) ≈ 1.4 mT and a($1\times{}^{1}H$) ≈ 0.2 mT.

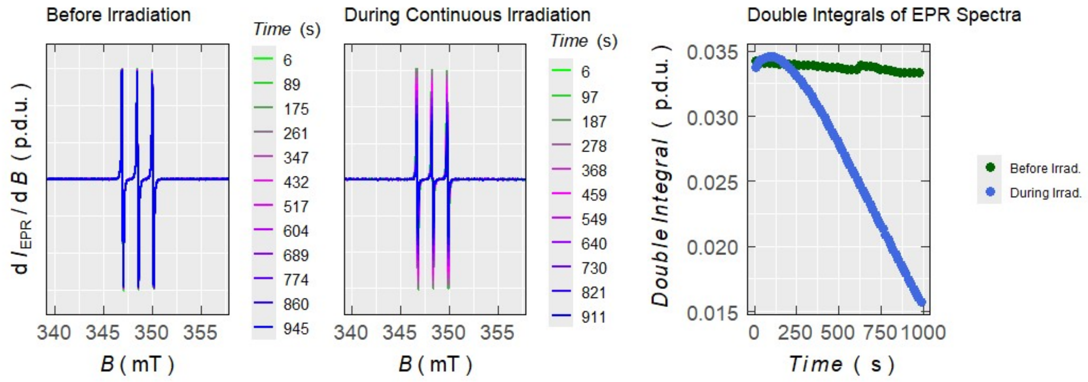

Figure S2-12: Time series EPR spectra recorded during the first 1000 s of the solution of TEMPO ($c = 0.2$ mM) with **BODIPY-Cl** ($c = 16$ mM) in PhH, conditioning in the dark (left) as well as during the continuous irradiation by 525 nm LED (middle). In addition, comparison between double integrals vs time, for the conditioning and for the irradiation, is presented for clarity. As can be seen, neither the position of the EPR spectrum ($g_{iso} = 2.0073$) nor the hyperfine splitting constant (a ≈ 1.55 mT) are changed. Therefore, all spectra correspond to aminoxyl, and no additional persistent radicals are formed or present upon irradiation.

Absorption spectra of solutions of **CH_3-BODIPY-Cl** in MeOH, with or without TEMPO, were recorded over time upon irradiation with the green light (525 nm) (Fig. S2-11). Chromatograms of the corresponding reaction mixtures before and after irradiation are shown in Fig. S2.12.

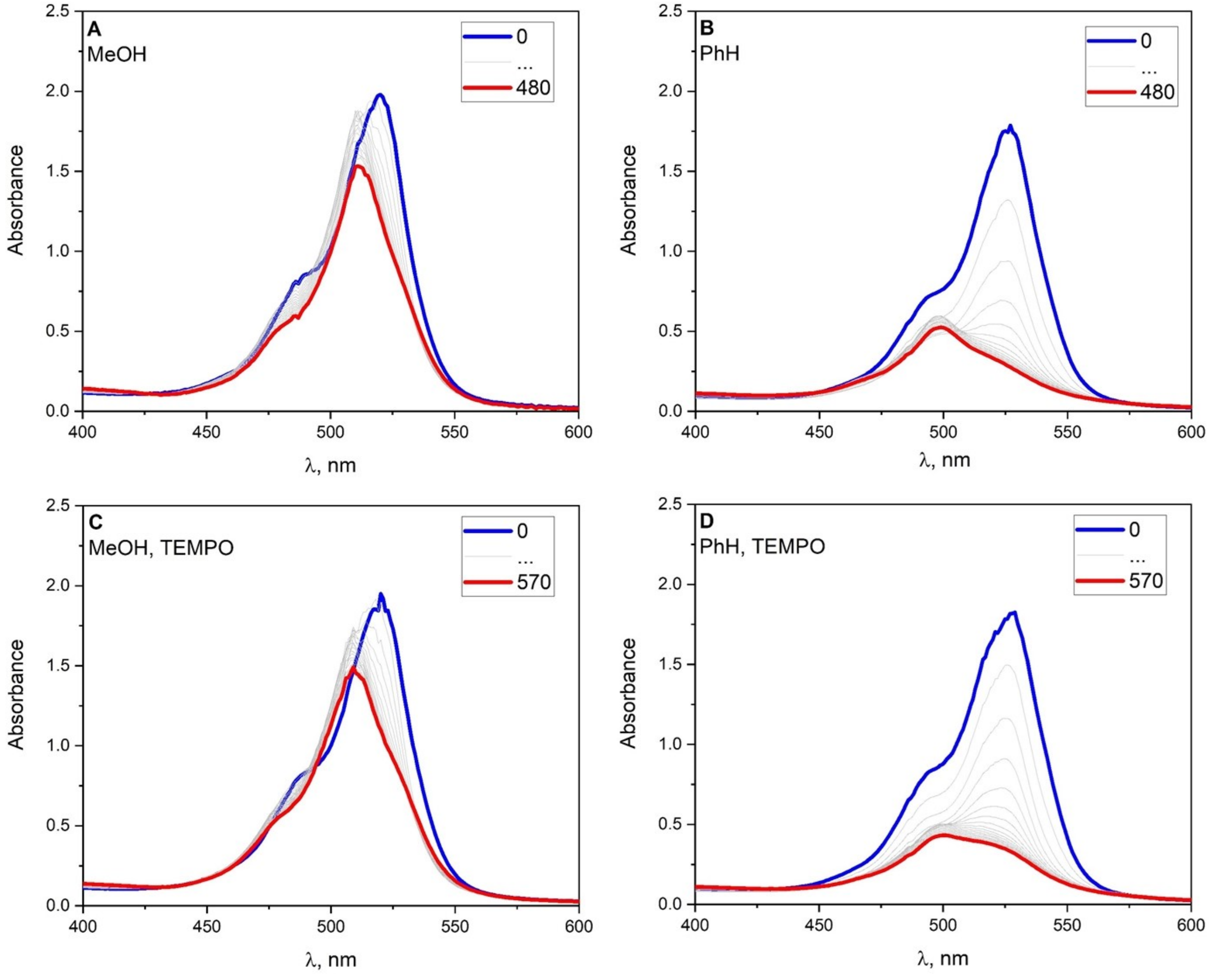

Figure S2-13: Absorption spectra of **CH_3-BODIPY-Cl** (c ~ 33 μM) in MeOH (A, C) or PhH (B, D) with (C, D) or without (A, B) TEMPO (15 eq.), before (blue), in time (grey), and after (red) irradiation with green light (525 nm).

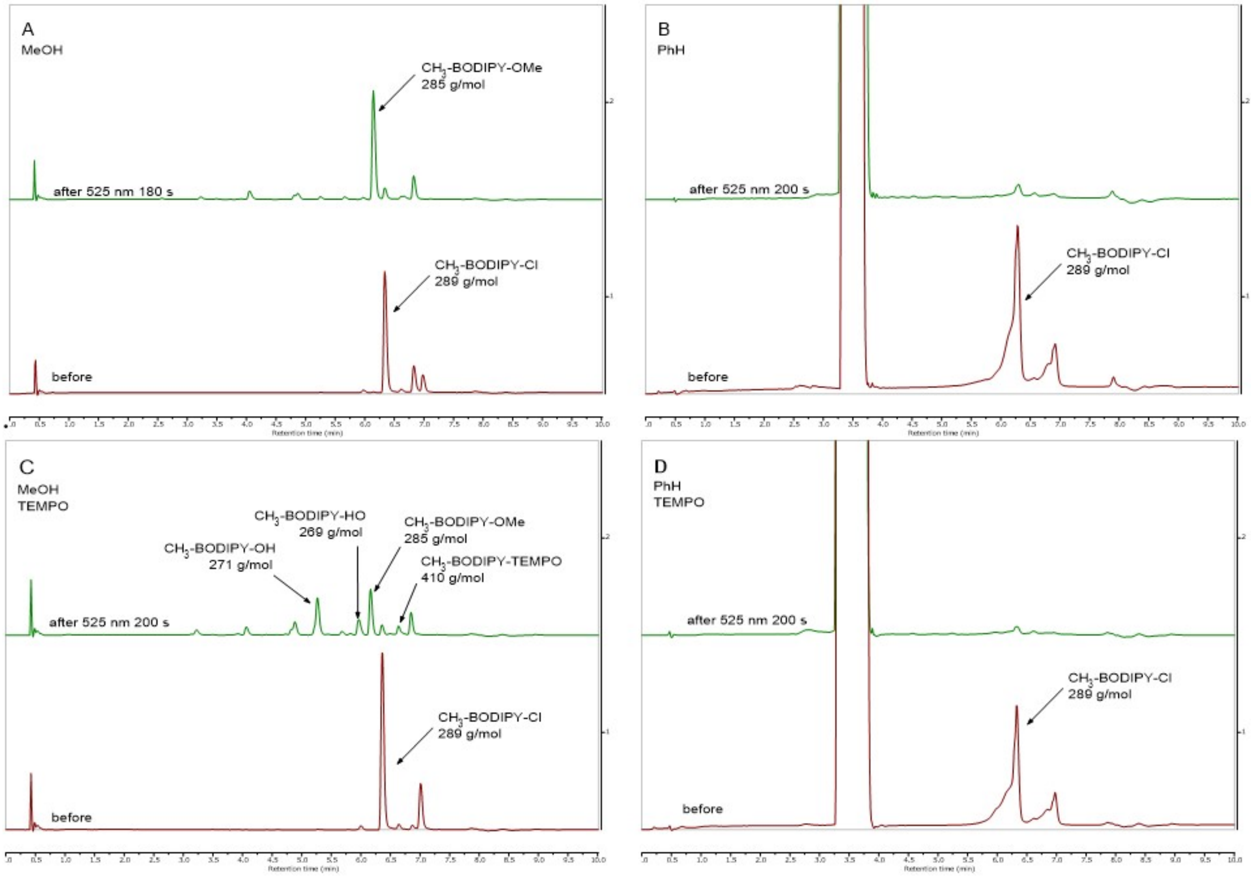

Figure S2-14: Chromatograms from UV-VIS detector (at 525 nm) of **CH_3-BODIPY-Cl** in MeOH (left, A, C) and PhH (right, B, D), before (red) and after (green) irradiation with 525 nm light. Top A, B: without TEMPO, bottom C, D: with TEMPO (15 eq.). Assignment of the compounds is based on the recorded mass spectra (ESI ionization)

Absorption spectra of solutions of **I-BODIPY-Cl** in MeOH, with or without TEMPO, were recorded over time upon irradiation with the green light (525 nm) (Figure S2-13). Chromatograms of the corresponding reaction mixtures before and after irradiation are shown in Figure S2-14.

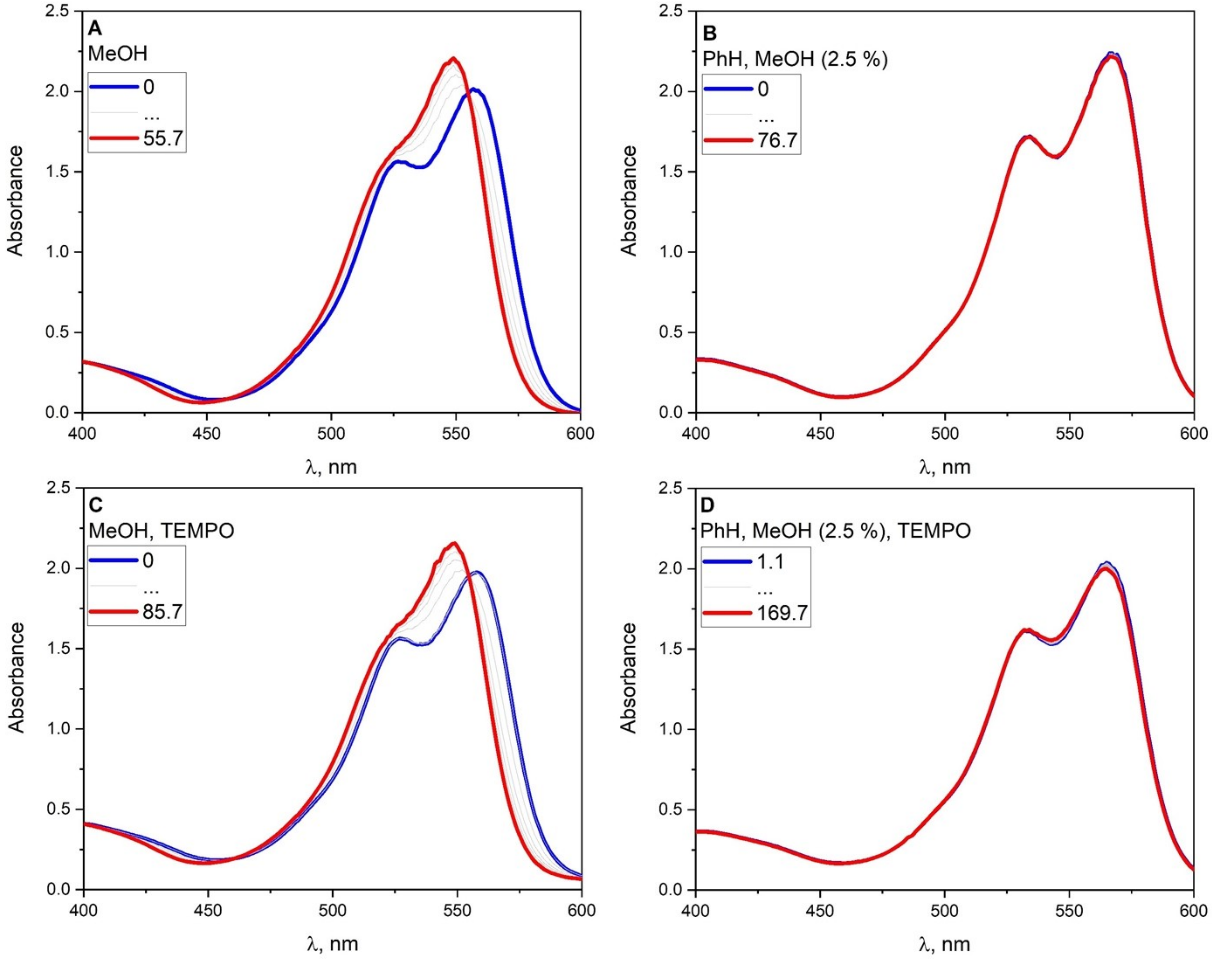

Figure S2-15: Absorption spectra of **I-BODIPY-Cl** (c ~ 37 μM) in MeOH (left, A, C) or PhH (right, B, D) with (bottom, C, D) or without (up, A, B) TEMPO (34 eq. (C) or 122 eq. (D)) before (blue), in time (grey), and after (red) irradiation with 525 nm light.

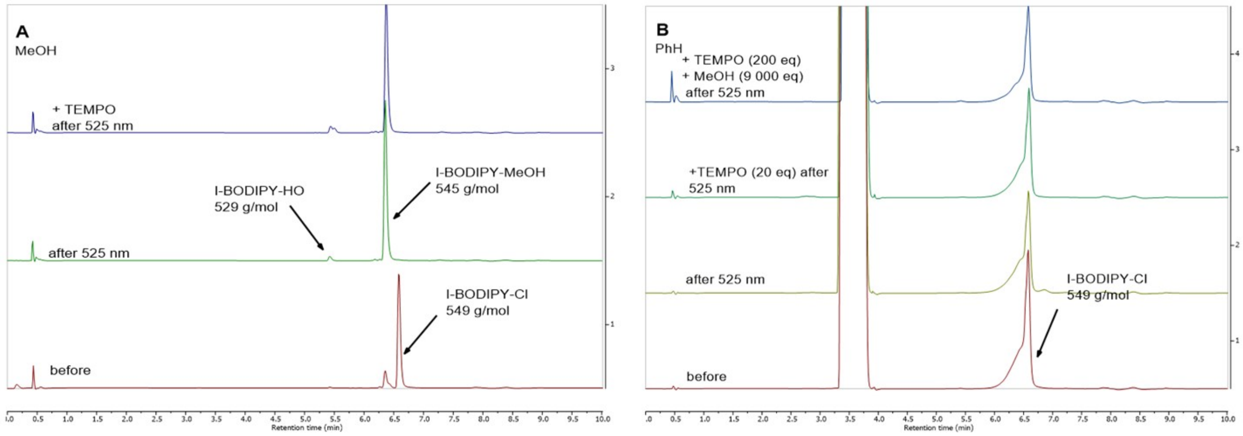

Figure S2-16: Chromatograms from UV-VIS detector (at 525 nm) of **I-BODIPY-Cl** in MeOH (A) and PhH (B). A: before (red) and after (blue with TEMPO (34 eq.), green without TEMPO) irradiation with 525 nm light. B: before (red), after (light green – no TEMPO, dark green – with TEMPO (34 eq.), blue – with 2.5 % of MeOH and TEMPO (122 eq.)) irradiation with 525 nm light. Assignment of the compounds is based on the recorded mass spectra (ESI ionization).

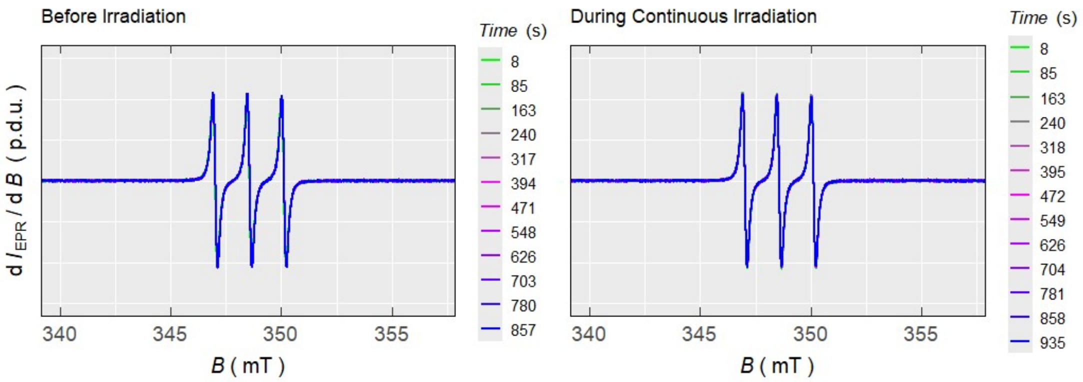

Figure S2-17: Time series EPR spectra recorded during the first 1000 s of a mixture of TEMPO (c = 0.2 mM) ans I-BODIPY-Cl (c = 16 mM) in PhH conditioning in the dark (left) as well as during the continuous irradiation by 525 nm LED (red). As can be seen, neither the position of the EPR spectrum (g_{iso} = 2.0075) nor the hyperfine splitting constant (a ≈ 1.56 mT) are changed. Therefore, all spectra correspond to aminoxyl, and no additional persistent radicals are formed or present upon irradiation

S2.3 Photoreactivity of BODIPY-TEMPO derivatives in benzene, methanol and *t*-BuOH

Light-induced reactivity of **BODIPY-TEMPO** in various solvents (MeOH, MeOH/PhH 1/1, PhH, *t*-BuOH) was studied using UV-Vis, ^{1}H NMR, EPR spectroscopies, and LC-MS. The results of the measurements gave evidence for the formation of TEMPO radical, along with **BODIPY-OH** and **BODIPY-CHO**.

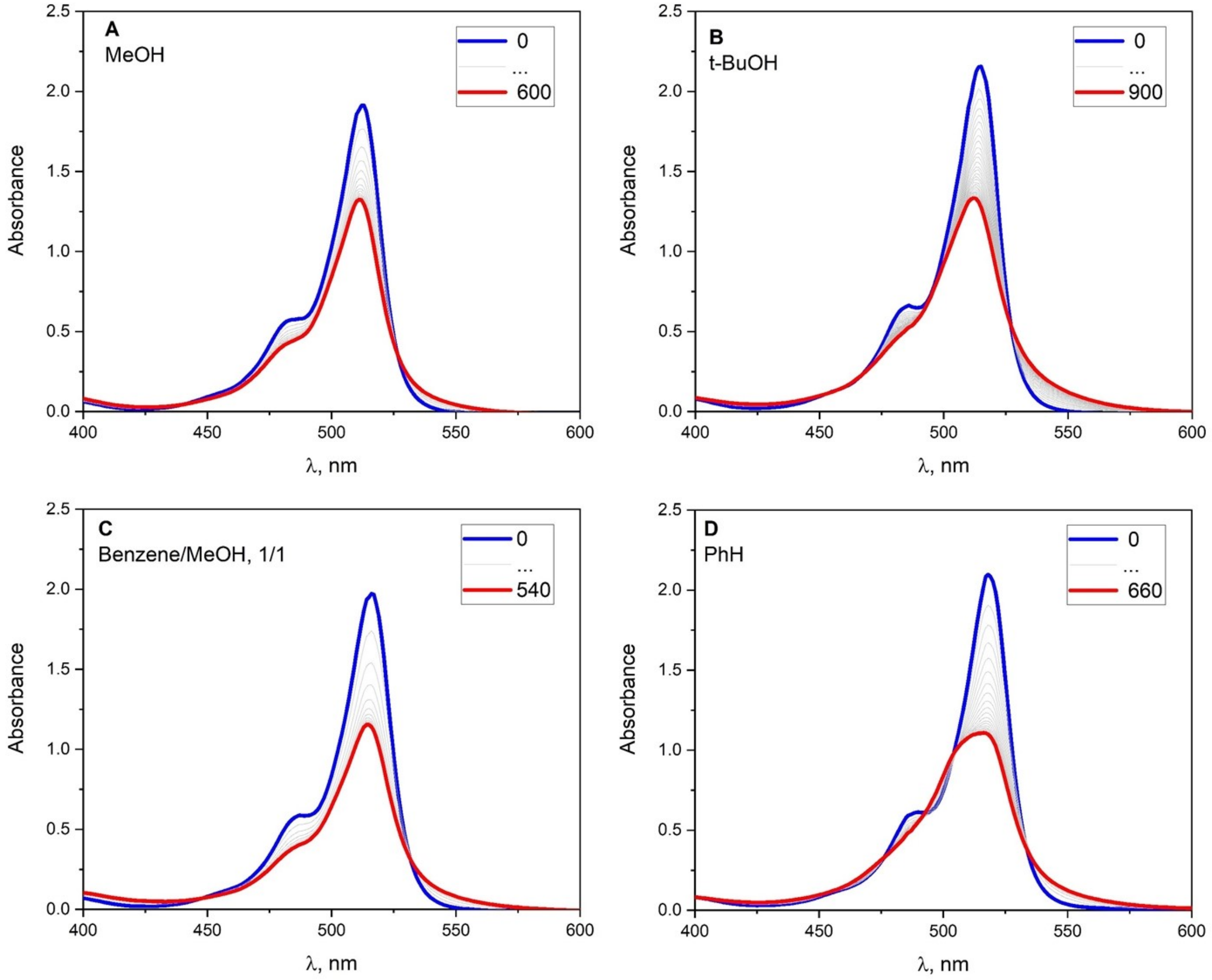

Figure S2-18: Absorption spectra of **BODIPY-TEMPO** in MeOH (A), *t*-BuOH (B), MeOH/PhH, 1/1 (C), and PhH (D) before (blue), in time (grey), and after (red) irradiation with green light (525 nm).

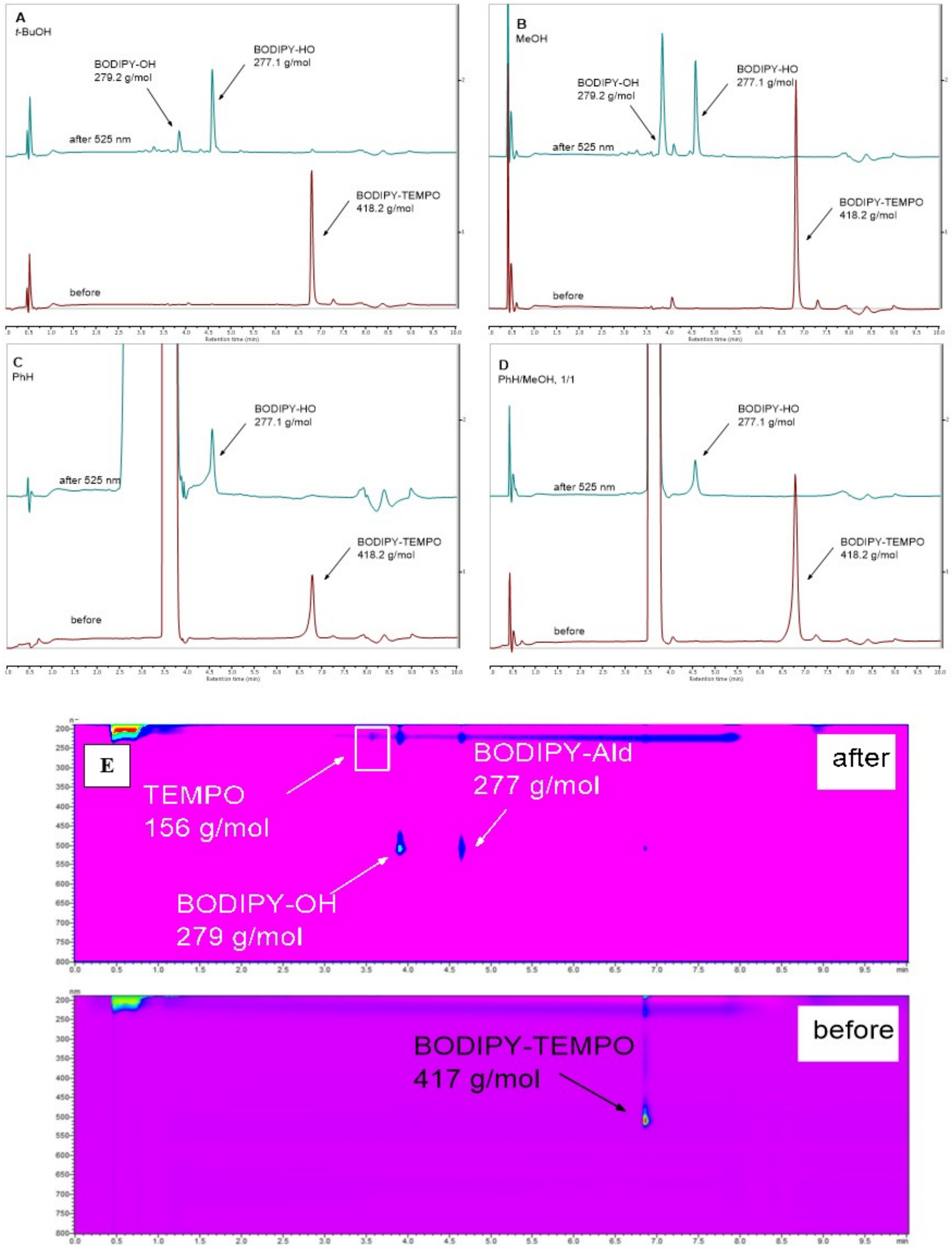

Figure S2-19: LC-MS studies. Chromatograms from UV-VIS detector (at 525 nm) of **BODIPY-TEMPO** in *t*-BuOH (A), MeOH (B), PhH (C), and MeOH/PhH, 1/1 (C) before (red) and after (blue) irradiation with 525 nm light. E: 3-D-Chromatograms of the reaction mixture A before and after irradiation.

BODIPY-TEMPO (1.3 mg) in 10.4 mL of solvent was charged into a 100 mL Erlenmeyer flask (optical path lengths, l ~ 3 mM), closed with a septum, and irradiated from the bottom with the 525 nm LEDs (18 min for full conversion according to LCMS). The solvents were removed from the reaction mixture under vacuum in the dark at r.t. The solid residual was dissolved in 0.4 mL of DMSO-*d6* and ^{1}H NMR spectra were recorded. ^{1}H NMR spectra showed the formation of **BODIPY-OH** and **BODIPY-CHO** in both PhH and MeOH, with a higher content of **BODIPY-OH** formed in MeOH.

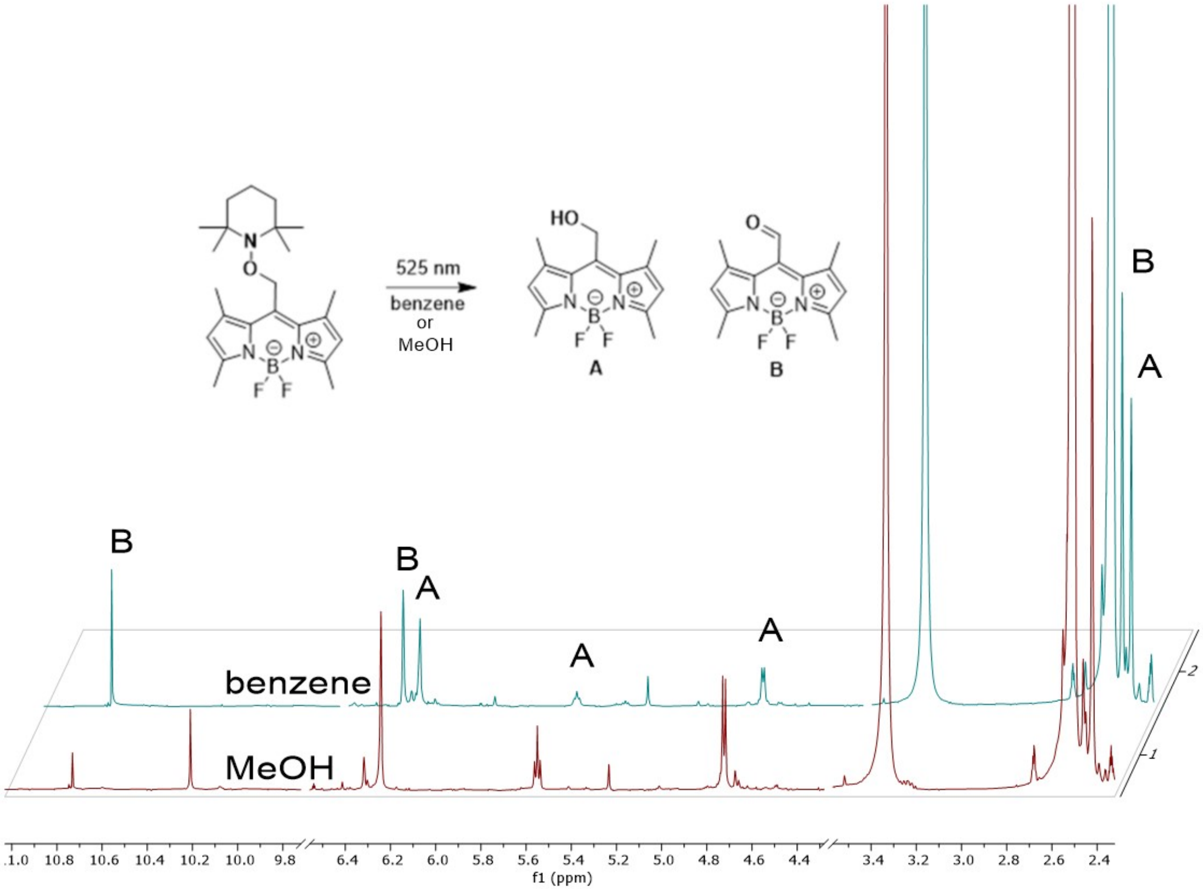

Figure S2-20: ^{1}H NMR spectra in DMSO-*d6* of **BODIPY-TEMPO** after irradiation with 525 nm light in MeOH (red) or PhH (blue).

Light-induced reactivity of **CH_3-BODIPY-TEMPO** and **I-BODIPY-TEMPO** in MeOH or PhH was studied using UV-Vis and EPR spectroscopy. The results of the measurements proved the formation of TEMPO radical upon photorelease.

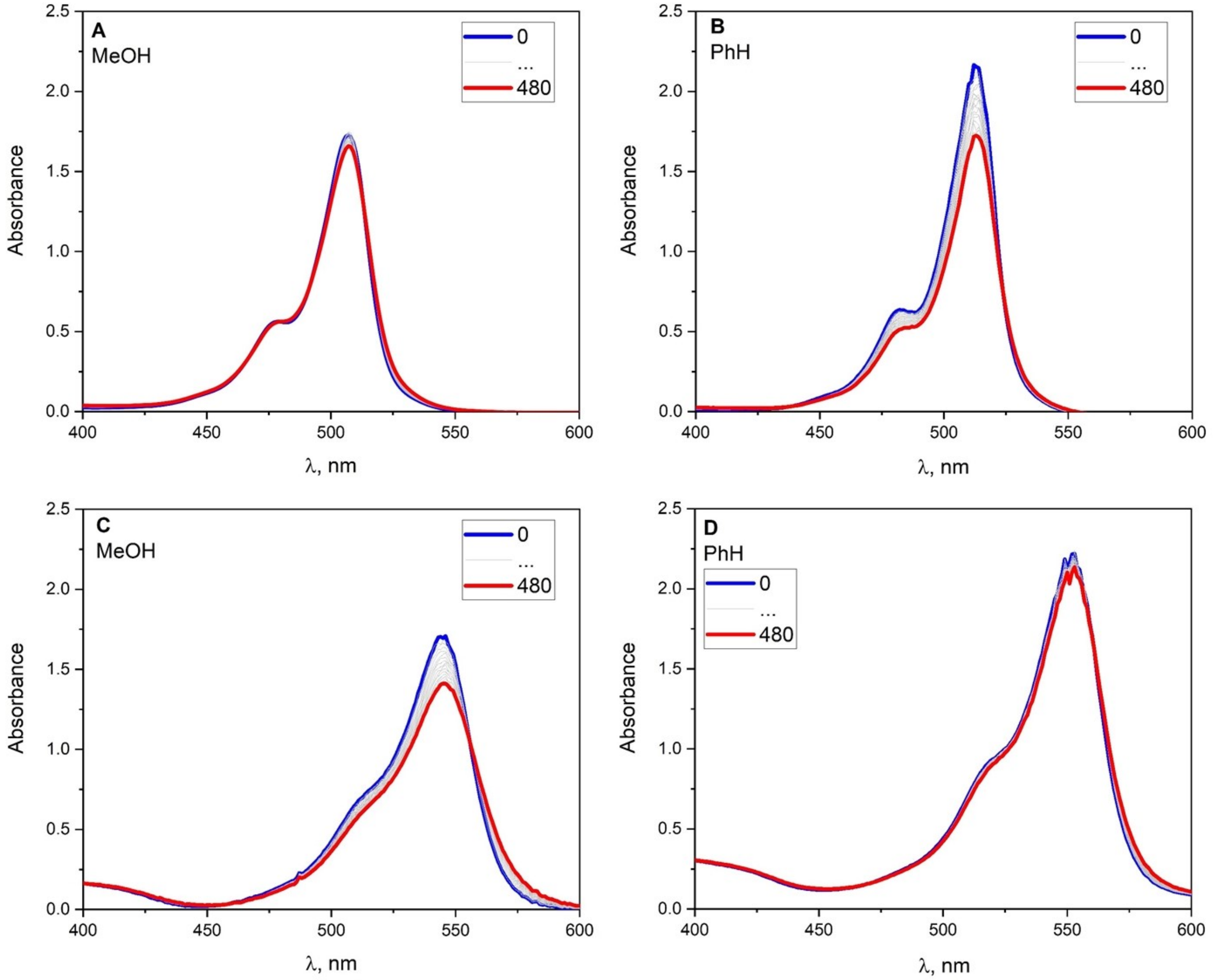

Figure S2-21: Absorption spectra of **CH_3-BODIPY-TEMPO** (A, B) and **I-BODIPY-TEMPO** (C, D) in MeOH (A, C) and PhH (B, D) before (blue), in time (grey), and after (red) irradiation with green light (525 nm).

EPR spectra of **X-BODIPY-TEMPO** compounds in MeOH or PhH measured upon irradiation demonstrated the photorelease of TEMPO. These data were also used to determine the efficiency (chemical and quantum yields) of TEMPO release and to compare its kinetics. Quantum yields of photorelease of TEMPO (Φ_r) were calculated from the presented kinetic curves (Fig. S2-20) and a value of photon flux of the light source used for EPR measurements. Solutions of **X-BODIPY-TEMPO** in PhH or MeOH (c = 1.7 × 10^{-4} M, ~ 50 uL) in capillaries (i.d. 1 mm, ~ 50 mm) were irradiated with 525 nm light (photon flux, I = 6.9 × 10^{-9} Einstein s^{-1}) for 1 000 or 2 000 s. Φ_{TEMPO} was calculated according to the equation shown below, where Δc is the change of concentration in M; t is time in s; V is the volume of the reaction mixture in dm^3; I is photon flux in Einstein s^{-1}.

$$\Phi_{rel} = \frac{\Delta c}{t}\frac{V}{I}$$

The abovementioned photon flux was determined using I-BODIPY-Cl as a chemical actinometer. Optically matched methanolic solutions of I-BODIPY-Cl were prepared in the dark due to the high

light sensitivity of this compound. Sealed capillaries were irradiated in the EPR setup for varying durations, then opened in the dark and analysed by LC-MS to follow the photoconversion of I-BODIPY-Cl to I-BODIPY-OMe with the quantum yield of photorelease (44.5%).[2]

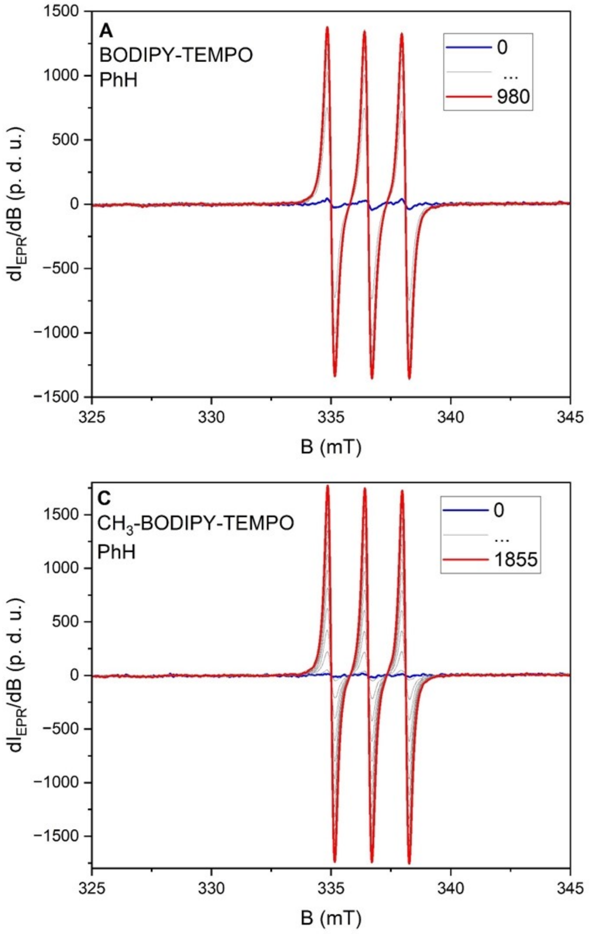

Figure S2-22: EPR spectra of **X-BODIPY-TEMPO** compounds in MeOH or PhH before (blue), in time (grey), and after (red) irradiation with green light (525 nm). A: **BODIPY-TEMPO** in PhH, B: **BODIPY-TEMPO** in MeOH, C: **CH_3-BODIPY-TEMPO** in PhH, D: I**-BODIPY-TEMPO** in PhH.

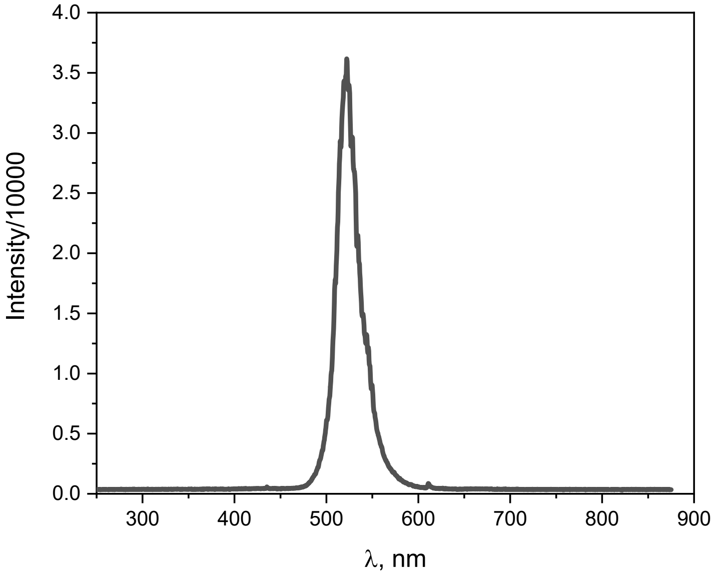

Figure S2-23: The emission spectra of LED light source.

S3. Polymers

Synthetic procedures for the preparation of *poly*-IBOA (Tab. S3.1, **e1-4**):

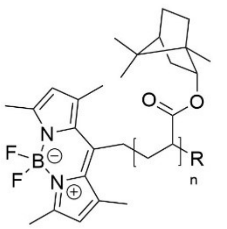

The reaction vessel (Schlenk tube, 10 mL or glass vial, 20 mL) was charged with **BODIPY-Cl** (0.02 – 0.08 mol%), 2-(dodecylthiocarbonothioylthio)-2-methyl-propionic acid (a RAFT agent) (0 or 0.4 mol%) and IBOA monomer (Tab. 3.1). The suspension was sonicated (1 min) and then stirred until a clear solution was formed (30 min). The reaction mixture was degassed under an argon atmosphere (3 x freeze-pump-thaw method (fpt) or 15 min bubbling). The reaction mixture was irradiated with green light (525 nm) at air cooling (20 °C). The polymers were isolated by precipitation from $CHCl_3$ (3 mL, 3 times) with MeOH (22 mL), followed by centrifugation and decantation. The polymers were dried under vacuum for 3 days.

Procedure for the ^{1}H NMR kinetic measurement (Tab. 3.1, **e-5**):

A Schlenk tube (10 mL) was charged with **BODIPY-Cl** (1.4 mg, 0.005 mmol, 0.04 mol%), RAFT (16.8 mg, 0.05 mmol, 0.4 mol%), and IBOA monomer (2.8 mL, 13.27 mmol). The suspension was sonicated (1 min). The tube was closed with a septum, and the reaction mixture was stirred until a clear solution was formed (30 min). The reaction mixture was degassed using the fpt method three times under an argon atmosphere. NMR tubes (9 pcs) were closed with septa, flushed with Ar for 5 min and charged with the reaction mixture (50 μL) under an inert argon atmosphere. Each tube was placed in front of the LED (530 nm, Fiber-Coupled LED, 1000 mA, SMA, Thorlabs connected to T-Cube LED Driver at 1000 mA) and irradiated for a defined time (0, 0.25, 0.5, 0.55, 1, 2, 3, 5, 11 h). A solution of 1,3,5-trioxane (5 mg) in $CDCl_3$ (0.5 mL) was added to the reaction mixtures and left for 1 h until the polymer was dissolved in all tubes. NMR spectra were recorded. Polymers formed in the reaction mixtures irradiated for 5 and 11 h were isolated by 3 times precipitation from DCM (0.5 mL) with MeOH (5 mL), followed by centrifugation and decantation. The polymers were dried under vacuum for 3 days.

^{1}H NMR (400 MHz, Chloroform-*d*) δ 4.63 (br s, 1H, CH), 2.27 (br s, 1H, CH), 2.06 – 1.35 (m, 7H, 2 x CH_2, 2 x $C\underline{H_A}H_B$, CH), 1.24 – 1.06 (m, 2H, 2 x $CH_A\underline{H_B}$), 0.96 (s, 3H, $C\underline{H_{3A}}CH_{3B}$), 0.83 (br s, 6H, $CCH_{3A}C\underline{H_{3B}}$, CH_3).

^{13}C NMR (101 MHz, Chloroform-*d*) δ 173.5 (COO), 81.2 (CHO), 48.8 (C), 47.0 (C), 45.2 (CH), 41.8 (CH_{poly}), 39.0 (CH_2), 36.5 (CH_{2poly}), 34.0 (CH_2), 27.3 (CH_2), 20.3 (2 x CH_3), 12.0 (CH_3).

IR-spectra: ṽ 1040 (m), 1155 (s), 1244 (m), 1370 (w), 1390 (w), 1455 (m), 1475 (w), 1728 (s, br), 2879 (m, br), 2934 (s, br), 2952 (s, br) cm^{-1}.

Fluorescence ($CHCl_3$): λ_{max} = 511 nm.

Table S3-1: Reaction conditions for the preparation of *poly*-IBOA and kinectic study.

	IBOA mL	BODIPY-Cl mg/mL	RAFT[a] mg/mL	Setup vessel/degassing /λ_{irr}	t_{irr} h	yield %	Mn g/mol	Đ	Φ_{fl}[b] %
**e-1a**	1	1	0	vial/bubbl./525 nm	7	14	313000	2.4	41
**e-1b**	1	1	6			17	11600	1.6	43
**e-2a**	1	1	0	Schlenk tube/fpt/525 nm	17	19	372000	2.4	
**e-2b**	1	1	6			32	17300	2.3	
**e-2c**	1	0.5	6			47	24600	1.4	
**e-3a**	1	1	0	Schlenk tube/fpt/525 nm	6	11	295000	2.3	
**e-3b**	1	1	0	Schlenk tube/bubbl./525 nm		11	314100	2.3	
**e-4a**	1	0.75	6	Schlenk tube/fpt/525 nm	17	33	15400	2.2	49
**e-4b**	1	0.5	6			44	21300	2.0	56
**e-4c**	1	0.25	6			40	20100	1.5	67
**e-5a**	0.05	0.5	6	NMR tube/fpt/530 nm	11	69[c]	34100	2.0	0
**e-5b**				(high-power LEDs)	5	62[c]	32600	1.8	27
**e-5c**					3	46[c]	27200	1.9	37

Characteristics of *poly*-IBOA (molar mass, dispersity Đ, and Φ_{fl}).

[a] Concentration of the RAFT agent; [b] measured in $CHCl_3$ with **BODIPY-OAc** as a standard, [c] Conversion calculated from NMR spectra

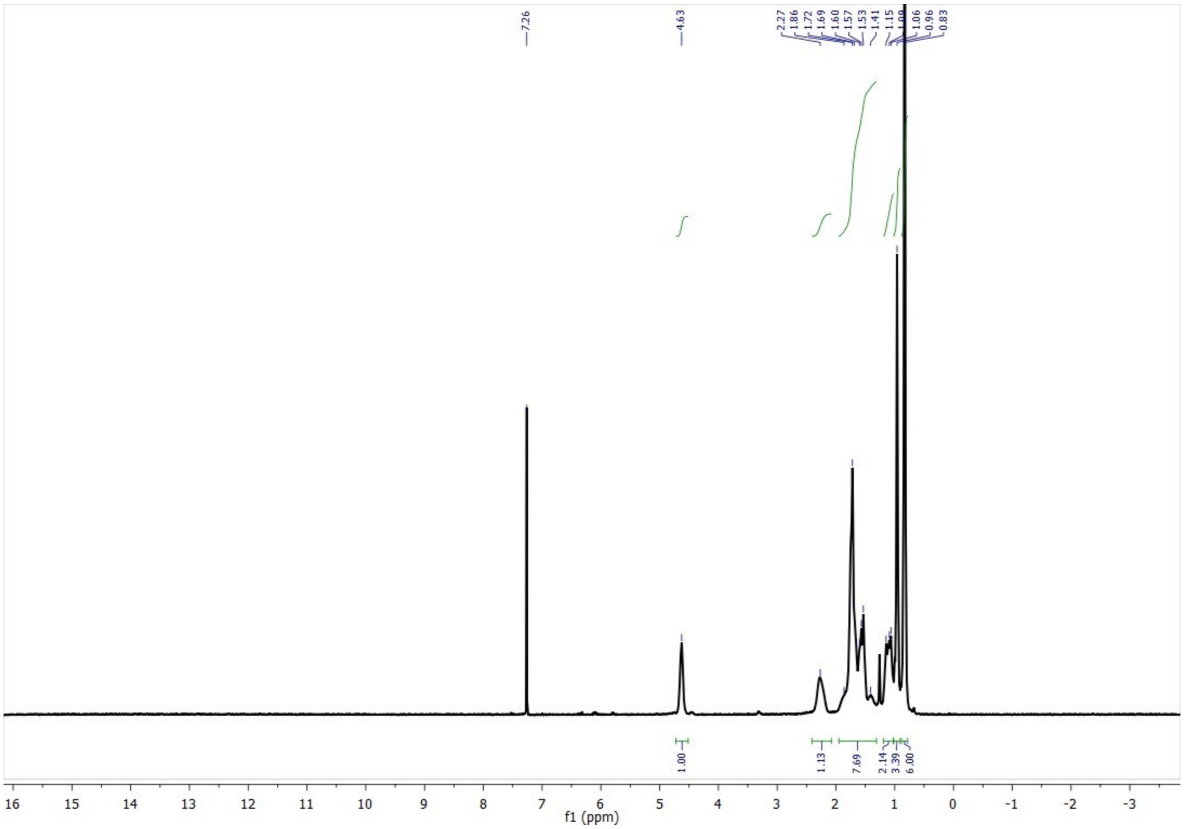

Figure S3-1: ^{1}H NMR spectrum of *poly*-IBOA in $CDCl_3$.

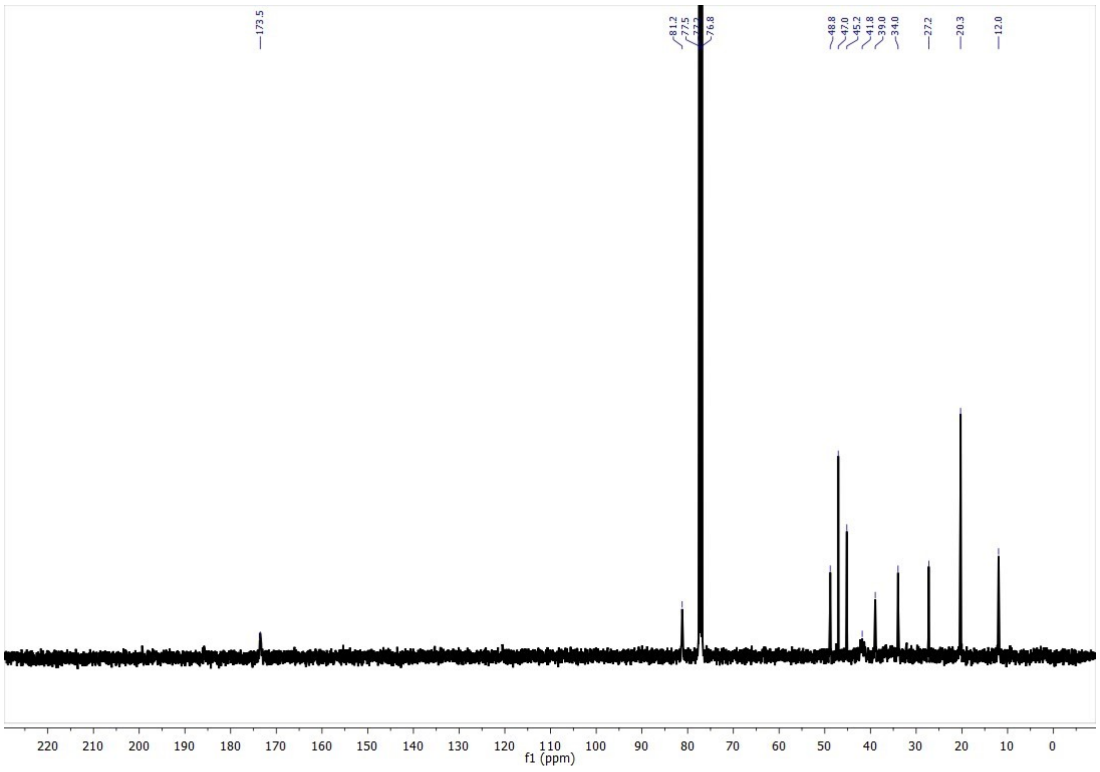

Figure S3-2: ^{13}C NMR spectrum of *poly*-IBOA in $CDCl_3$.

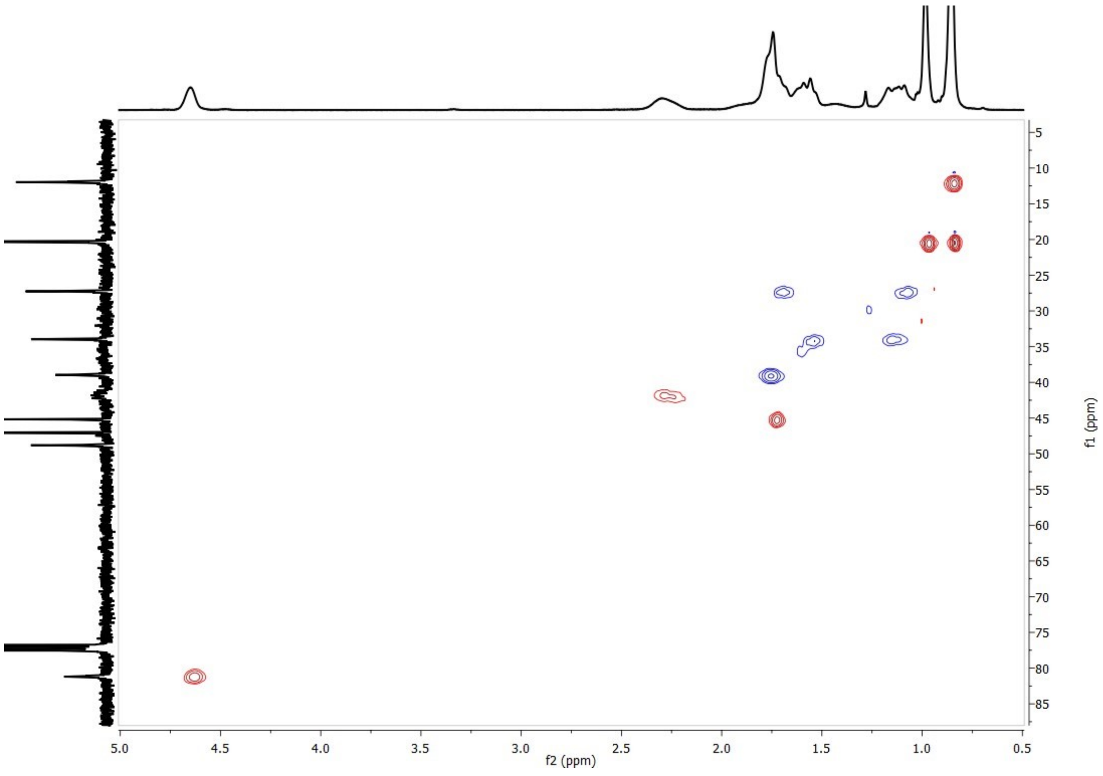

Figure S3-3: HSQC NMR spectrum of *poly*-IBOA in $CDCl_3$.

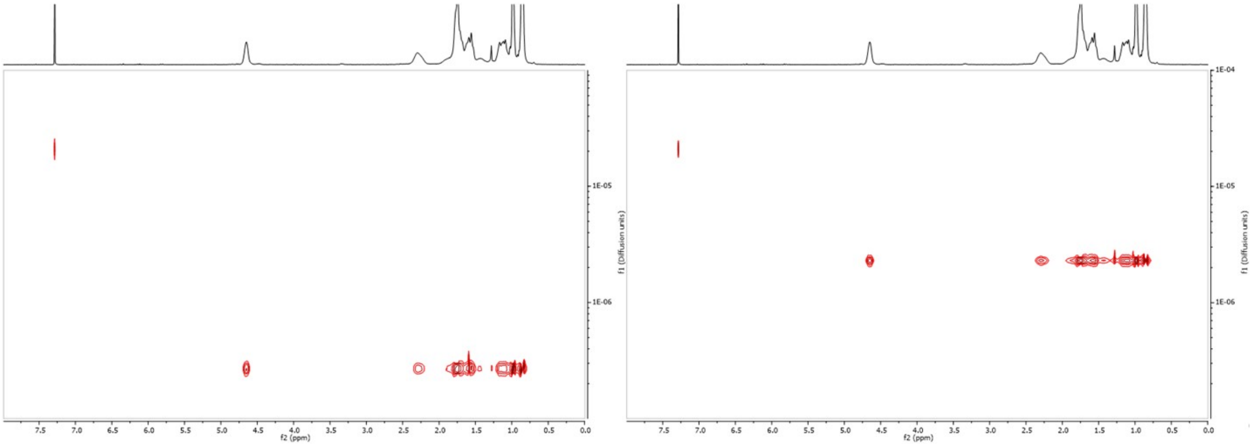

Figure S3-4: DOSY NMR spectra of *poly*-IBOA in $CDCl_3$ prepared using free radical chain polymerization (**e-1a**, left) and RAFT polymerization (**e-1b**, right).

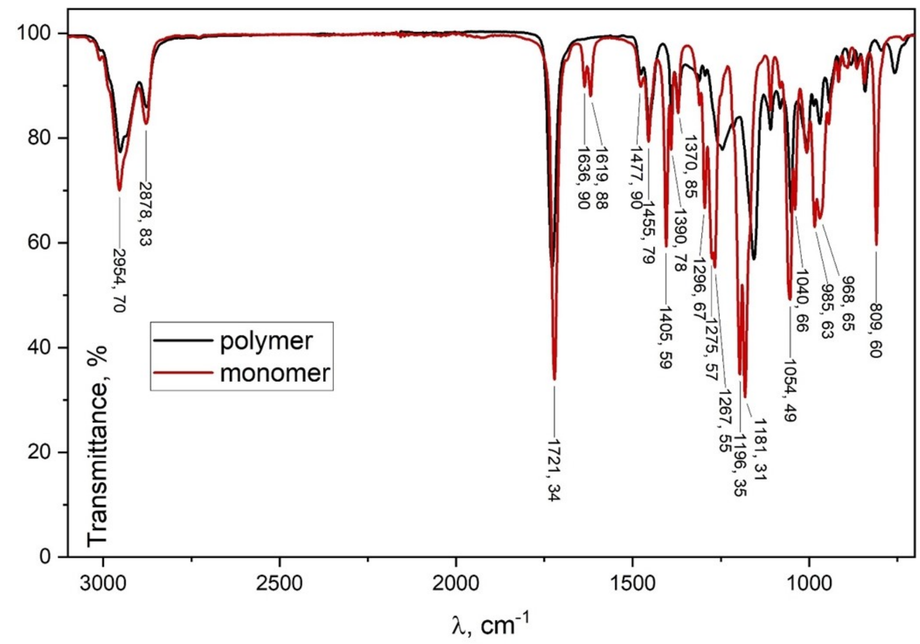

Figure S3-5: IR spectra of films of IBOA monomer (black) and *poly*-IBOA (red).

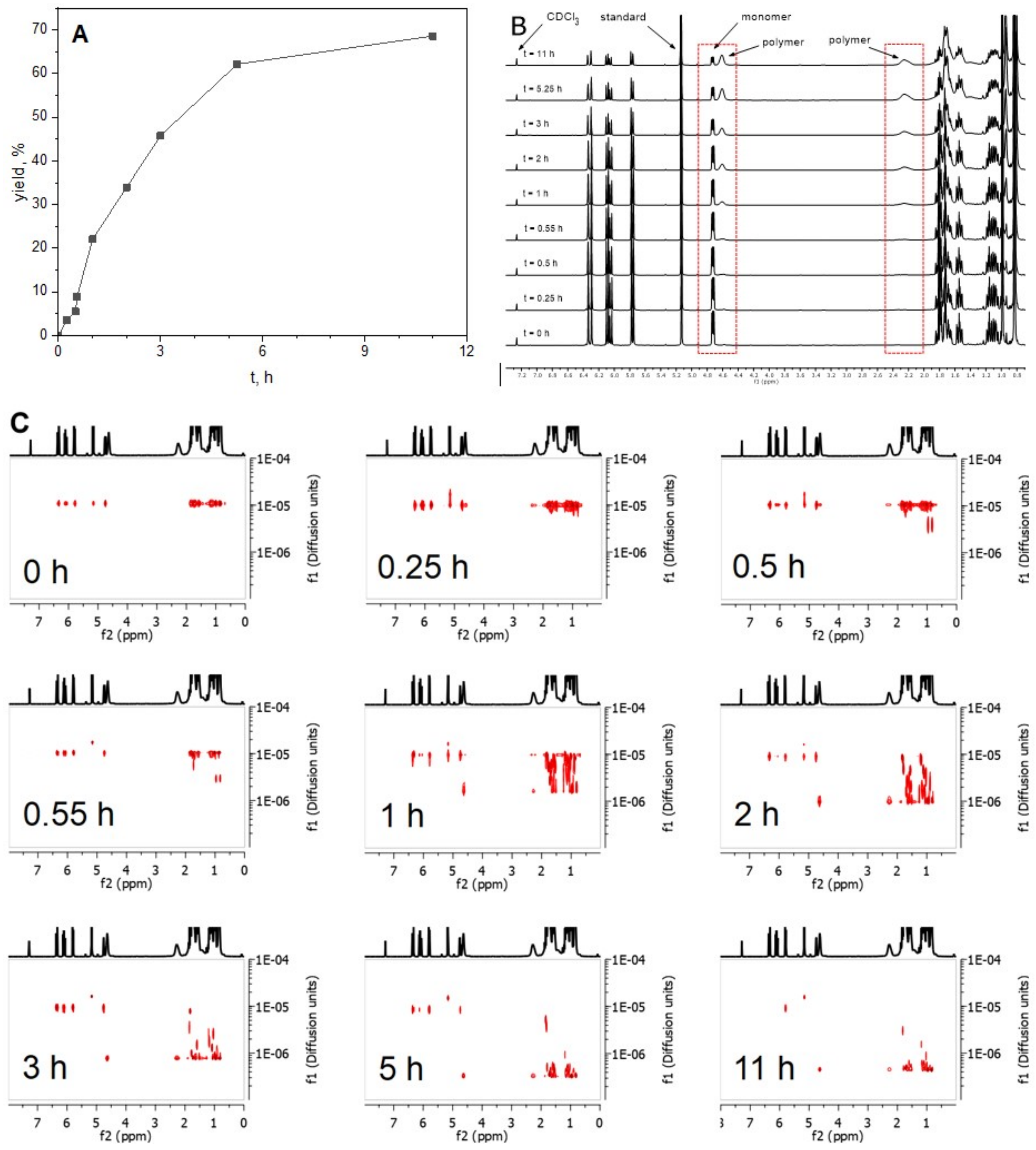

Figure S3-6: A. Concentration of *poly*-IBOA over time upon irradiation with green light (530 nm). B. ^{1}H NMR spectra recorded over time. C. 2D-DOSY NMR spectra recorded over time.

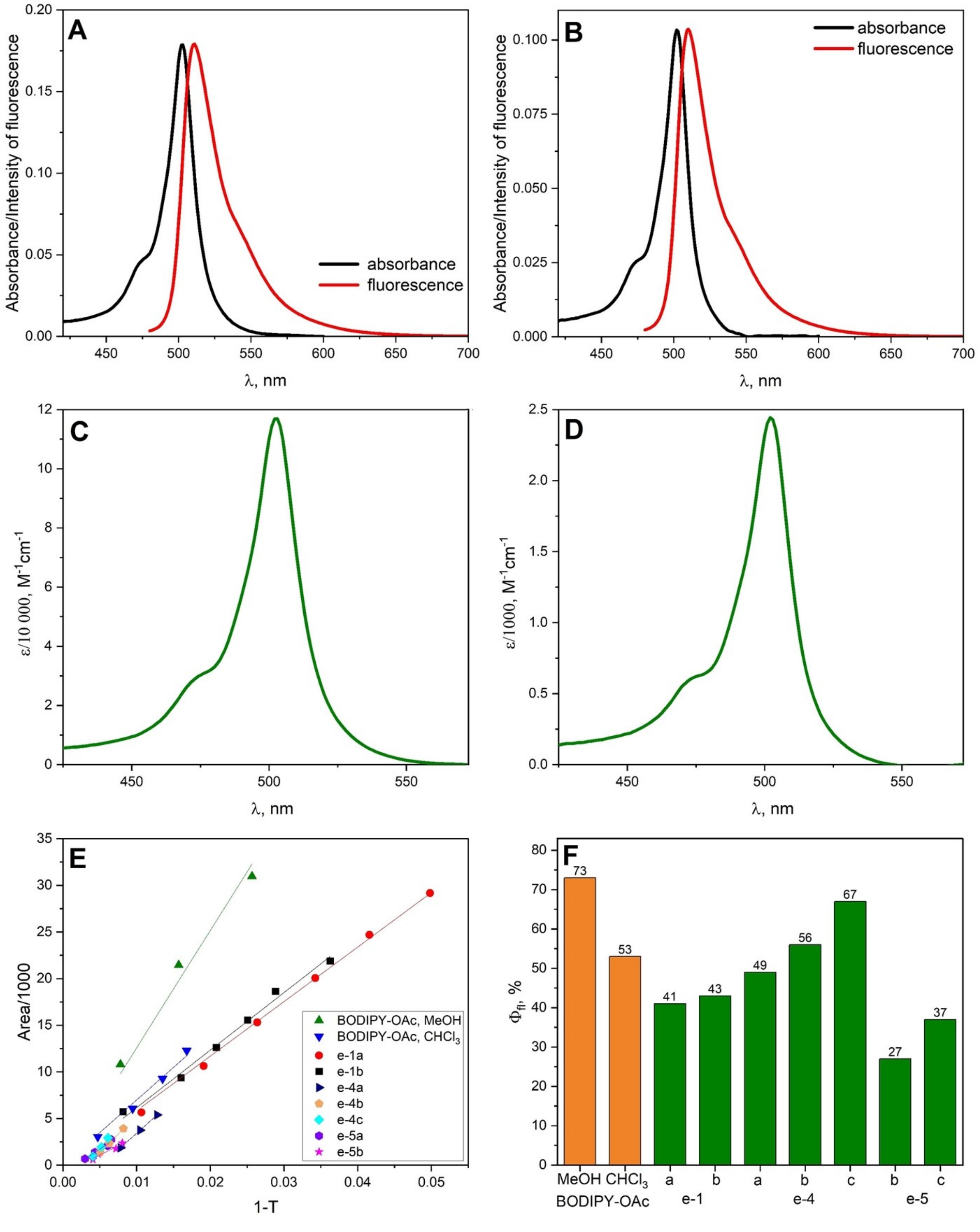

Figure S3-7: A,B: Absorption (black) and normalized intensity of fluorescence (red) spectra of *poly*-IBOA **e-1a** (A, c = 1.5 μM) and **e-1b** (B, c = 41 μM) in $CHCl_3$. C,D: Molar extinction coefficients ε ($M^{-1}cm^{-1}$) of *poly*-IBOA **e-1a** (C) and **e-1b** (D). E: Plot for determination of Φ_{fl} for

the standard compound **BODIPY-OAc** (in MeOH or $CHCl_3$) and *poly*-IBOA **e-1-5** in $CHCl_3$. F: corresponding Φ_{fl} values.

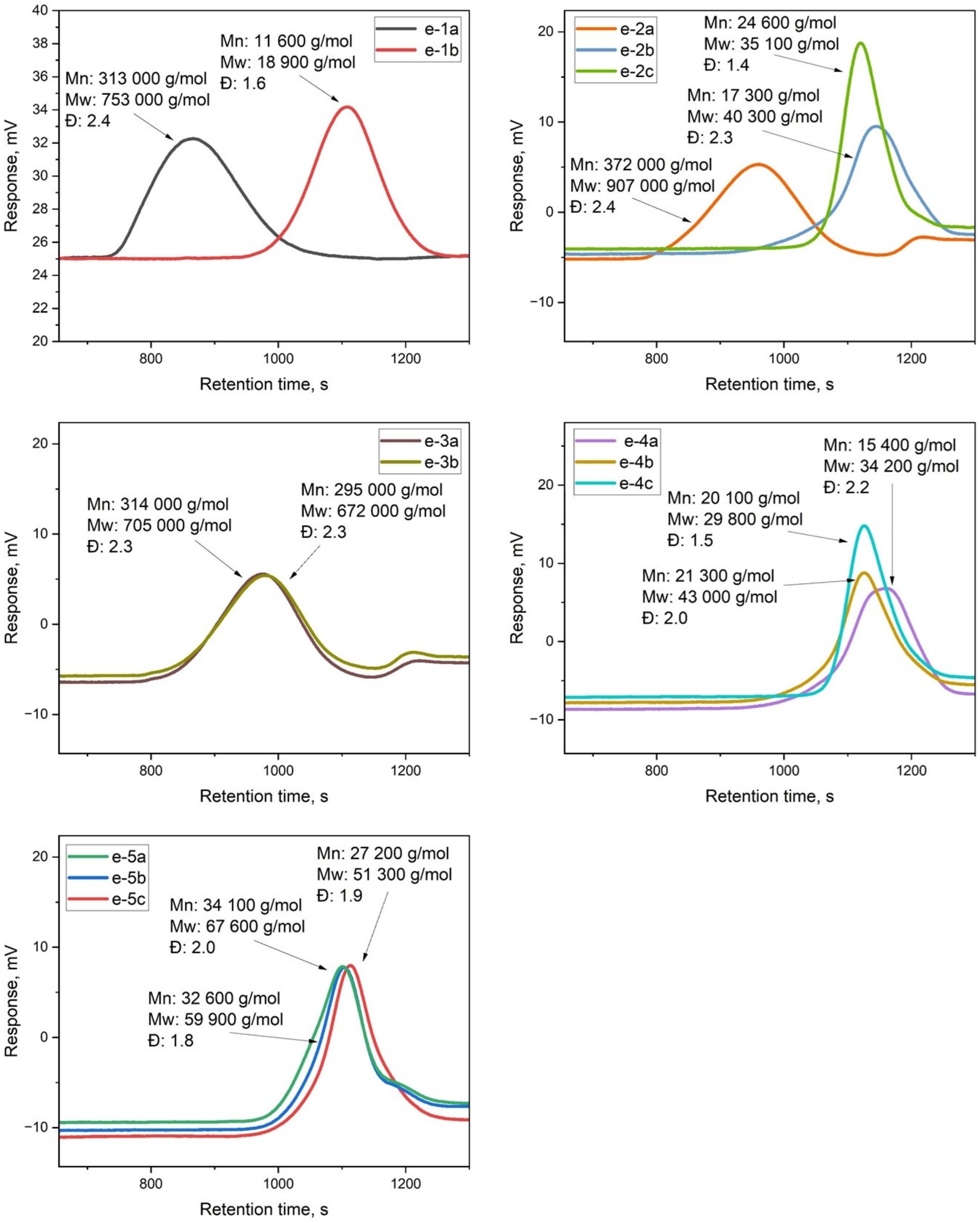

Figure S3-8: GPC chromatograms for *poly*-IBOAs **e-1-5**.

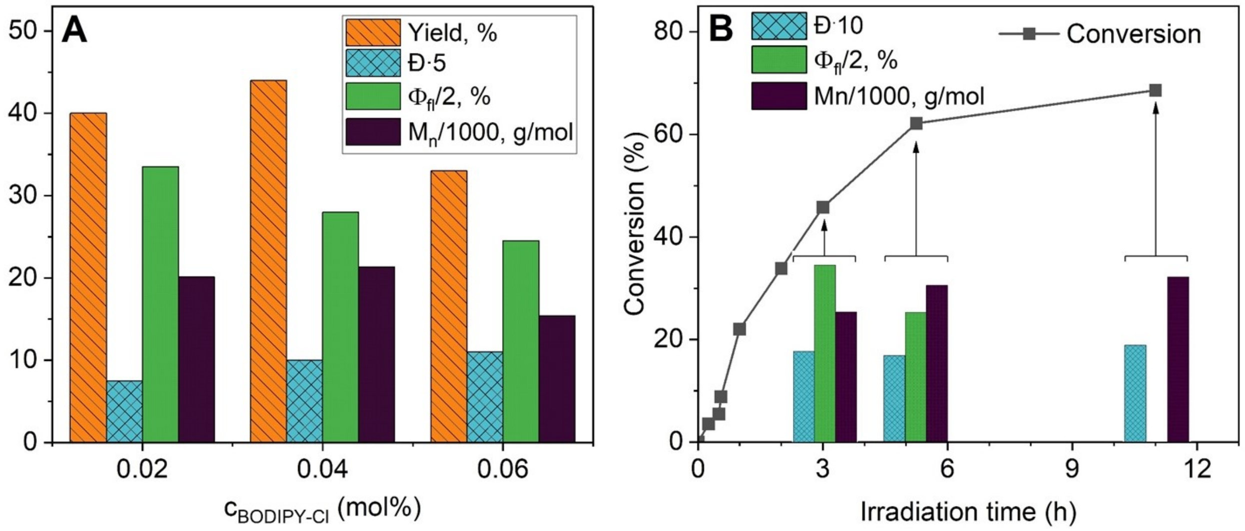

Figure S3-9: **A**. Properties of **e-4**; **B**. Kinetics of polymerization **e-5** of *poly*-IBOA: yield (orange), Đ (blue), Φfl (green), and M_n (violet).

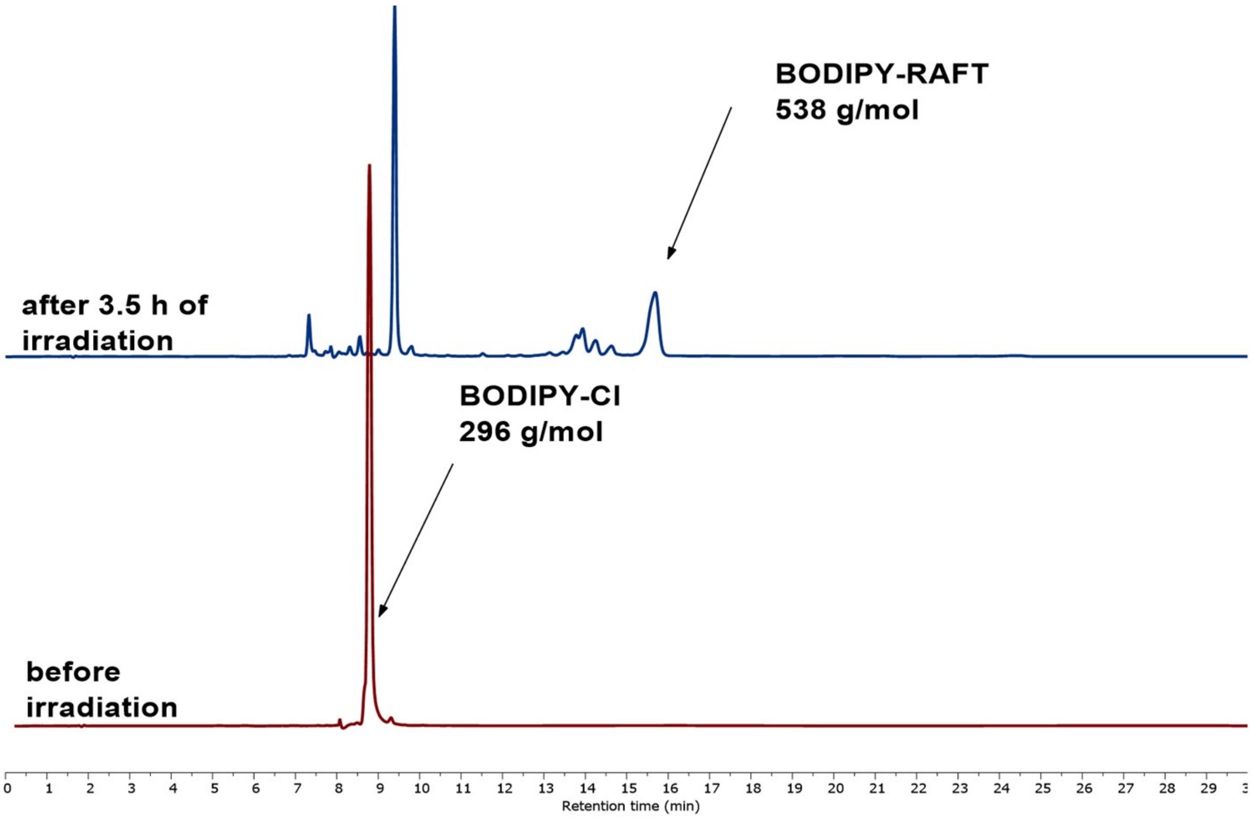

Figure S3-10: LC-MS studies. Chromatograms from UV-VIS detector (at 450-550 nm) of **BODIPY-Cl** (1 mg/mL) and **DDMAT** (12 mg/mL) in *toluene-d8* before (red) and after (blue) irradiation with 525 nm light. Assignment of the compounds is based on the recorded mass spectra (ESI ionization).

S4. References

1. M. Reinfelds, V. Hermanns, T. Halbritter, J. Wachtveitl, M. Braun, T. Slanina, A. Heckel, *ChemPhotoChem*, **2019**, 3, 6, 441–449.
2. T. Slanina, P. Shrestha, E. Palao, D. Kand, J. A. Peterson, A. S. Dutton, N. Rubinstein, R. Weinstain, A. H. Winter, P. Klán, *J. Am. Chem. Soc.* **2017**, 139, 15168–15175.
3. Bruker. 2025. "EPR Sofware/Xenone Software." https://www.bruker.com/en/products-and-solutions/mr/epr-instruments/epr-software/xenon.html
4. A Guide to Recording Fluorescence Quantum Yields, HORIBA UK Limited https://static.horiba.com/fileadmin/Horiba/Application/Materials/Material_Research/Quantum_Dots/quantumyieldstrad.pdf
5. https://refractiveindex.info/
6. F. Wilkinson, W. P. Helman, A. B. Ross, *J. Phys. Chem. Ref. Data*. **1993**, 22, 113–262.
